# Surface-Enhanced Raman Detection of the CO_2_ Moisture Swing

**DOI:** 10.1021/acsaenm.5c00716

**Published:** 2025-11-05

**Authors:** Javier Mendez Lozoya, Estrella Solis Mata, J. Jesus Velazquez Salazar, Alondra Hernandez Cedillo, Miguel Jose Yacaman, Jennifer L. Wade

**Affiliations:** † Department of Applied Physics and Materials Science, 3356 Northern Arizona University, Flagstaff, Arizona 86011, United States; ‡ Center for Materials Interfaces in Research and Applications, Northern Arizona University, Flagstaff, Arizona 86011, United States; § Instituto Tecnológico de San Luis Potosí, Tecnológico Nacional de México, Av. Tecnológico s/n, Soledad de Graciano Sánchez, San Luis Potosí 78437, Mexico; ∥ Steve Sanghi College of Engineering, Department of Mechanical Engineering, Northern Arizona University, Flagstaff, Arizona 86011, United States

**Keywords:** CO_2_ capture, moisture swing, anion exchange, SERS, Raman spectroscopy, Ni coated Ag NWs, in situ analysis

## Abstract

The development of scalable, energy-efficient carbon dioxide (CO_2_) capture technologies is critical for achieving net-zero emissions. Moisture swing (MS) sorbents offer a promising alternative to traditional thermal regeneration methods by enabling reversible CO_2_ binding through humidity-driven ion hydrolysis. In this study, we investigate the anion speciation dynamics in two classes of MS materials—an anion-exchange resin with a bicarbonate anion and activated carbon impregnated with potassium bicarbonate salt—using both sorption measurements and in situ surface-enhanced Raman spectroscopy (SERS). Ni-coated Ag nanowires were employed as SERS substrates to enhance signal intensity and enable the real-time detection of carbonate (CO_3_^2–^), bicarbonate (HCO_3_^–^), and hydroxide (OH^–^) species under controlled humidity conditions in both air and nitrogen atmospheres. The results reveal humidity-dependent interconversion between anionic species with significant spectral shifts confirming the reversible hydrolysis reactions that drive the MS mechanism. Under humid conditions, we observed the depletion of bicarbonate signals and a concurrent increase in carbonate species, consistent with moisture-induced desorption of CO_2_. With the activated carbon samples, we further observed the formation of hydroxide. These findings not only validate the mechanistic models of humidity-driven anion exchange in MS sorbents but also demonstrate the practical potential of SERS as an operando diagnostic tool for monitoring CO_2_ capture media. The ability to resolve and semiquantitatively evaluate the reversible transformation of carbonate, bicarbonate, and hydroxide ions under realistic environmental conditions provides valuable insight for the rational design, performance optimization, and quality control of next-generation sorbent materials for direct air capture applications.

## Graphical abstract

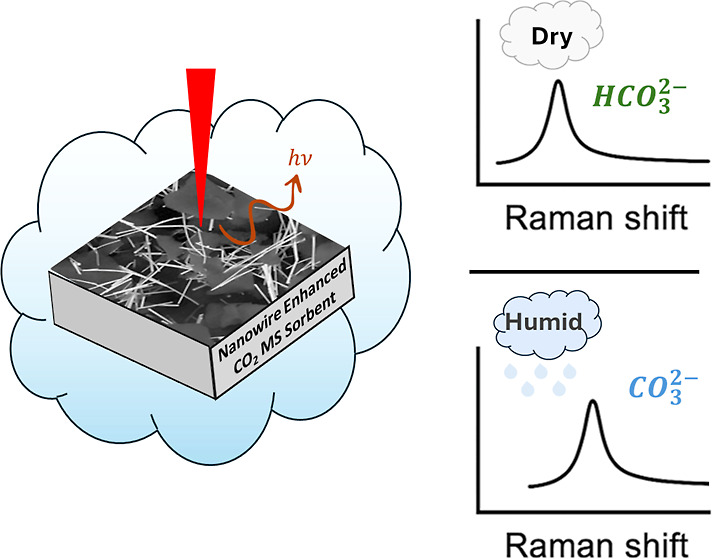

## 1 Introduction

Scalable and affordable carbon dioxide (CO_2_) capture from dilute gas streams has grown in emphasis over the past decade to achieve both negative greenhouse gas emissions^1,2^ and a circular carbon economy.^3^ Emerging commercial technology for the separation of CO_2_ from air, termed direct air capture (DAC), is based on hydroxide solvents^4^ or supported amine sorbents.^5^ The energetics of both approaches are dominated by the thermal energy required to regenerate the phase separating media and in turn concentrate the CO_2_ for subsequent compression and storage or use.^6^ The solid sorbent system has potential to drive down energetic costs through waste heat integration or renewable power heat pumps, as regeneration temperatures for these materials range from 80 to 120 °C versus the hydroxide system requiring >900 °C regeneration of the trapped CO_2_ species, solid CaCO_3_. However, the sorbent’s CO_2_ separation capacity is hampered by the thermal oxidative stability of the amine chemistry, limiting the amount of CO_2_ captured per unit of amine sorbent.^7–9^ Beyond the early commercial approaches, other promising technologies include the use of metal oxide sorbents, also requiring high-temperature regeneration (>900 °C),^10^ and amino acid solvents,^11^ also prone to thermal degradation.^12^

The work in this study examines an emerging family of CO_2_ separating materials that use hydration rather than heat to regenerate and concentrate CO_2_, the moisture swing (MS).^13–22^ All MS-responsive materials provide nanoconfined domains that constrain reactive anions like anion exchange resins,^13,19,20,22,23^ anion exchange membranes,^16^ quaternized graphene sheets,^17^ or with simple salt impregnated into microporous structures.^15,18,21^ Guided by DFT studies^23–25^ and MS measurements of CO_2_ sorption across a range of alkaline anions, the central hypothesis behind the MS separation is that anion hydrolysis varies with humidity. The CO_2_-based MS relies on the hydrolysis reactivity of the alkaline carbonate, CO_3_^2–^anion as a function of hydration, which is in turn controlled by the surrounding water activity i.e., relative humidity (RH) (eq 1).

1 $${CO}_{3}^{2-}\cdot m_{1}H_{2}O↔{HCO}_{3}^{-}\cdot m_{2}H_{2}O+{OH}^{-}\cdot m_{3}H_{2}O+\left(m_{1}-m_{2}-m_{3}-1\right)H_{2}O$$

In the above expression, the divalent carbonate can dissociate water from its hydration cloud (*m*_1_H_2_O) to form the conjugate monovalent bicarbonate (HCO_3_^–^·*m*_2_H_2_O) and hydroxide species (OH^–^·*m*_3_H_2_O). Notably, this hydrolysis becomes energetically favored at low hydration levels, where the monovalent anions are stabilized relative to the divalent carbonate. The amount of water released is dependent on the stoichiometric size of the hydration clouds (*m*_*i*_H_2_O), which is dictated by the affinity of water to the anion–cation pair and the affinity of water to the surrounding structure at a given level of water activity. In the presence of PCO_2_, the hydroxide will readily bind this acid gas to form a further bicarbonate anion (eq 2).

2 $${CO}_{2}+{OH}^{-}\cdot m_{3}H_{2}O↔{HCO}_{3}^{-}\cdot m_{2}H_{2}O$$

Taking eqs 1 and 2 together, the net chemical reaction of CO_2_ binding can be represented as eq 3, where the hydroxide species is treated as a transitory player.

3 $${CO}_{3}^{2-}\cdot m_{1}H_{2}O+{CO}_{2}↔2H{CO}_{3}^{-}\cdot m_{2}H_{2}O+\left(m_{1}-m_{2}-m_{3}-1\right)H_{2}O$$

The overall result is a net release of water upon binding of CO_2_, where *m*_1_-*m*_2_-*m*_3_-1 > 0. Consequently, introducing water to the system shifts eq 3 to the left, liberating CO_2_ and resulting in the MS in accordance with Le Chatlier’s principle. Under low PCO_2_, the equilibrium in eq 2 favors the hydroxide form. This latter point has been shown both through MS-based biocomponent isotherms^26,27^ and dynamic breakthrough column experiments,^23^ where total CO_2_ (de)­sorption matches the ion exchange capacity of the anion exchange resin when desorption proceeds under low, PCO_2_ < 1 Pa, and in alkaline electrolyte studies where hydroxide-exchanged anion exchange films remain in the OH- state only under an inert atmosphere (e.g., in UHP N_2_).^28,29^ Thus, it is the influence of both water activity (hydration) and PCO_2_ that dictates the anion speciation in these confined materials.

Most MS investigations have evaluated CO_2_ and H_2_O sorption through gas phase changes in water vapor and CO_2_. Fewer studies have experimentally interrogated the reactive species to validate the above mechanism. Two exceptions have been the use of solid-state NMR to probe the carbonate/bicarbonate species after equilibration at different levels of moisture.^17,30^ Nicotera et al. examined the MS mechanism in trimethylammonium-functionalized graphene oxide using ^13^C NMR, and there they saw the depletion of HCO_3_^–^ and formation of CO_3_^2–^ when equilibrating to 400 ppm^13^ CO_2_ at various humidity levels.^17^ This study did not explore the formation of OH^–^, which was the initial anion in their material. Yang, Singh, and Schaefer 2018 similarly used ^13^C NMR methods to monitor anion speciation from a polystyrene, divinylbenzene cross-linked anion exchange resin,^30^ also initialized in the hydroxide form. Yang et al. also observed the formation of HCO_3_^–^ after binding with CO_2_; however, the investigators did not see the emergence of a CO_3_^2–^ phase upon the addition of humidity. Rather they observed the formation of hydroxide, suggesting a different reactive pathway than the one proposed by eq 3. Details of the PCO_2_ were not reported in this latter case. A clear understanding of the MS reactive mechanism is essential in informing future material advancements that aim to increase CO_2_ capacity, sorption kinetics, or material stability.

The goal of this work is to examine the anion speciation in two classes of MS-responsive materials: a commercial anion exchange resin, IRA900, and an activated carbon impregnated with potassium carbonate salts using in situ Raman spectroscopy. The use of an environmental chamber interfacing with a Raman laser and detector enables a step change in water activity surrounding the sorbent materials while in the presence (∼400 ppm CO_2_ in air) or absence of CO_2_ (in ultrahigh purity N_2_). This in turn enables the detection of emerging or depleting anion phases as they react with the surrounding atmosphere.

Raman spectroscopy, based on inelastic light scattering, enables qualitative identification of molecular species and quantitative determination of analyte concentrations, making it ideal for evaluating the reactive anion of anion-exchange resins.^31–39^ Surface-Enhanced Raman Spectroscopy (SERS) leverages localized surface plasmon resonance and surface plasmon polaritons on nanostructured Ag, Au, or Cu substrates to enhance electromagnetic fields by several orders of magnitude, enabling trace-level detection and real-time monitoring of adsorption–desorption dynamics critical to assessing resin performance.^40–48^

In the present study, we utilize Ni-coated Ag nanowires (NWs) as SERS-active substrates. These nanowires, previously characterized in earlier work,^49^ combine the strong plasmonic activity of silver with the magnetic functionality of nickel. The one-dimensional anisotropic geometry of the nanowires facilitates the generation of localized electromagnetic “hot spots,” especially at junctions and tips, leading to significantly enhanced Raman signal intensities. The Ni shell provides magnetic responsiveness, enabling facile manipulation and potential substrate reusability and thus enhancing experimental reproducibility. Additionally, the large surface area and high structural integrity of Ni coated Ag NWs promote effective analyte adsorption and long-term stability, rendering them excellent candidates for sensitive and reproducible SERS-based detection.

## 2 Methods

### 2.1 Materials

The MS materials explored in this study include a divinylbenzene cross-linked polystyrene anion exchange resin with trimethylammonium groups charge-balanced in the chloride form (IRA900-Cl), and potassium bicarbonate-impregnated activated charcoal (AC-KHCO_3_). All raw sorbent materials were purchased from Sigma-Aldrich: IRA-900-Cl (Amberlite), activated charcoal (Spectrum Chemicals), and potassium bicarbonate salts (ACS reagent, >99%). The IRA900 resin is received as a 0.5–1 mm spherical bead. To enhance contact between the resin and a nanowire, the resin was cryomilled using a SPEX SamplePrep 6870 cryogenic milling machine, with a milling cycle of 5 min, followed by a 2 min cooling period in liquid nitrogen to dissipate heat buildup. This cycle was repeated six times. To activate the IRA900-Cl to a MS responsive state, the chloride must be ion-exchanged to a reactive form. For this study, the IRA900-Cl was ion-exchanged using two 24 h washes in 0.1 M KHCO_3_ solution at a ratio of 15 mL liquid:20 mg resin. Excess potassium salts were rinsed from the resin using deionized (DI) water. The concentration of exchanged Cl^–^ to HCO_3_^–^ was measured by using Hach chloride strips in the ion-exchange solution. After two washes the net ion exchange capacity was 3.0 ± 0.1 mmol/g resin, where the numerator represents the dry mass of the IRA900-Cl after sitting in room air for 24 h (∼15% RH, 20 °C). Both the bicarbonate-exchanged resin (*IRA*900-HCO_3_) and the as-received chloride resin (IRA900-Cl) were evaluated in this study, with the latter acting as a control to compare results to the reactive form. The porous activated charcoal samples were wet impregnated with 1.0 M KHCO_3_ following the procedure detailed by Zheng et al.^18^ The Ni-coated Ag nanowires used for the surface enhancement were prepared using methods described elsewhere.^49^

### 2.2 Surface-Enhanced Sample Preparation

We impregnated 50 μL of a cryomilled IRA900-Cl or *IRA*900-HCO_3_ solution with 50 μL of a silver–nickel core–shell nanowire (Ni-coated Ag NW) suspension. To ensure a homogeneous mixture suitable for deposition and analysis, we dispersed both components in deionized water. In a similar approach, we impregnated salt-treated *i*-KHCO_3_ samples with 50 μL of a Ni-coated Ag NWs suspension. We used a 1 M KHCO_3_ solution as the dispersion medium to retain the reactive salt species on the carbon surface during impregnation. The resulting suspensions were homogenized using a vortex mixer, deposited onto aluminum boats, and air-dried overnight prior to further analysis (Figure 1).^36^

**Figure 1:**
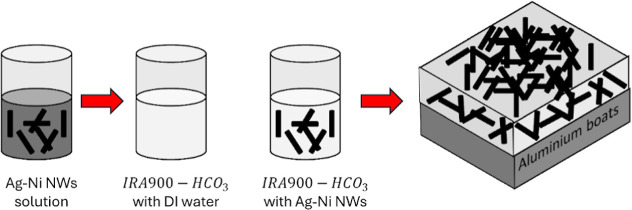
Diagram illustrating the fabrication process of SERS substrates.

### 2.3 Material Characterization

The morphology of the Ni-coated Ag nanowires (NWs) and IRA900-HCO_3_ resin was characterized using a Phenom ProX scanning electron microscope (SEM) equipped with a backscattered electron (BS) detector, operated at an accelerating voltage of 10 kV, and a Talos F200i scanning transmission electron microscope equipped with a bright field detector and operated at 200 kV. Elemental composition of the nanowires was examined via energy-dispersive X-ray spectroscopy (EDS) mapping.

Further, the moisture-swing-based CO_2_ and H_2_O countersorption of the sorbents (void of nanowire) was characterized by comparing sorption behavior relative to the speciation observed in Raman measurements. We monitored this mixed gas sorption using a modified Netzsch STA 449 F3 Jupiter instrument, which combines thermogravimetric analysis (TGA), differential scanning calorimetry, and outlet gas analysis (Li-7000 infrared H_2_O/CO_2_ analyzer) with control of the gas composition in an open flow configuration. This contrasts with most MS characterizations explored in a closed flow configuration where only changes in gas composition are controlled and monitored.^15,19,21,27^ Experiments were performed under 25 °C isothermal conditions, and step-changes in humidity were controlled using a modular humidity generator system (MHG32, Prohumid). Sorbents were equilibrated for 5 h at 25 °C in a 20% RH humidified stream of 400 ppm of CO_2_ in ultrahigh-purity nitrogen (N_2_) with a total dry gas flow of 200 sccm. At 5 h intervals, the water activity was stepped between 95 and 20% RH, and the corresponding changes in sorbent mass, heat flow, and gas composition were recorded. Total sorbed CO_2_ over time was calculated from the integration of the outlet CO_2_ gas concentration signal relative to the final 60 min baseline of the 5 h segment. In contrast, total sorbed H_2_O over time was calculated from the net mass change measured with the TGA microbalance, corrected for the mass of CO_2_ (de)­sorbed in each step. Further details on the instrumentation, gas flow configuration, and mass balance calculations are described elsewhere.^26^

### 2.4 In Situ Raman Environmental Chamber

Moving beyond gas and mass sorption analyses, we directly probed the compositional changes of the sorbent materials under varying environmental conditions using surface-enhanced Raman scattering (SERS) spectroscopy. In situ Raman measurements were taken over time in open-flow, dry, and humidified conditions using either compressed air, which contained approximately 450 ppm CO_2_, or ultrahigh-purity nitrogen (N_2_). Prior to introducing water vapor, the dry gas passed through the line connected to second mass flow controller at a rate of 100 sccm for 3 h, drying the sorbent sample. The outgoing feed gas was then directed to a Li-850 CO_2_/H_2_Ogas analyzer, where the concentrations of H_2_O (in PPT) and CO_2_ (in PPM) were measured. After drying, the gas was switched to flow through MFC1, which directed the flow into a humidifier, where the gas was sparged into DI water using an aeration stone. Figure 2 shows the schematic configuration used to make the Raman measurements using Horiba XploRA PLUS equipment. Raman spectra were acquired using a red laser with a wavelength of λ = 638 nm, an 1800 cm^–1^ grating, and a 10× Nikon objective lens. The incident laser power was set to 10 mW, and spectra were collected over the range 400–2000 cm^–1^ for the polymer resins. This range was selected to minimize potential photothermal damage associated with the use of a red laser excitation source. Lower-wavenumber regions, typically below 400 cm^–1^, are more susceptible to fluorescence interference and thermal degradation, particularly in carbon-based or salt-impregnated materials. By restriction of the measurement range, the structural integrity of the sample was preserved while capturing the most relevant vibrational modes for compositional and structural characterization. Raman spectra of the AC-KHCO_3_ samples were acquired using a green laser excitation source with a wavelength of λ = 532 nm. The measurements were performed with a grating of 1800 cm^–1^ and a 10× Nikon objective lens, providing adequate spatial resolution for surface analysis. A laser power of 10 mW was employed to ensure sufficient signal intensity while avoiding thermal degradation of the sample. Spectral data were collected over the range of 400–3800 cm^–1^, enabling comprehensive analysis of both low- and high-frequency vibrational modes associated with the carbon framework and surface-bound species. For each sample, 27 Raman spectra were recorded under both dry and humid conditions in air and nitrogen gas atmospheres. Averaging across these replicates enabled the generation of representative spectra for each condition, minimizing the impact of instrumental noise and sample variability on spectral interpretation.

**Figure 2:**
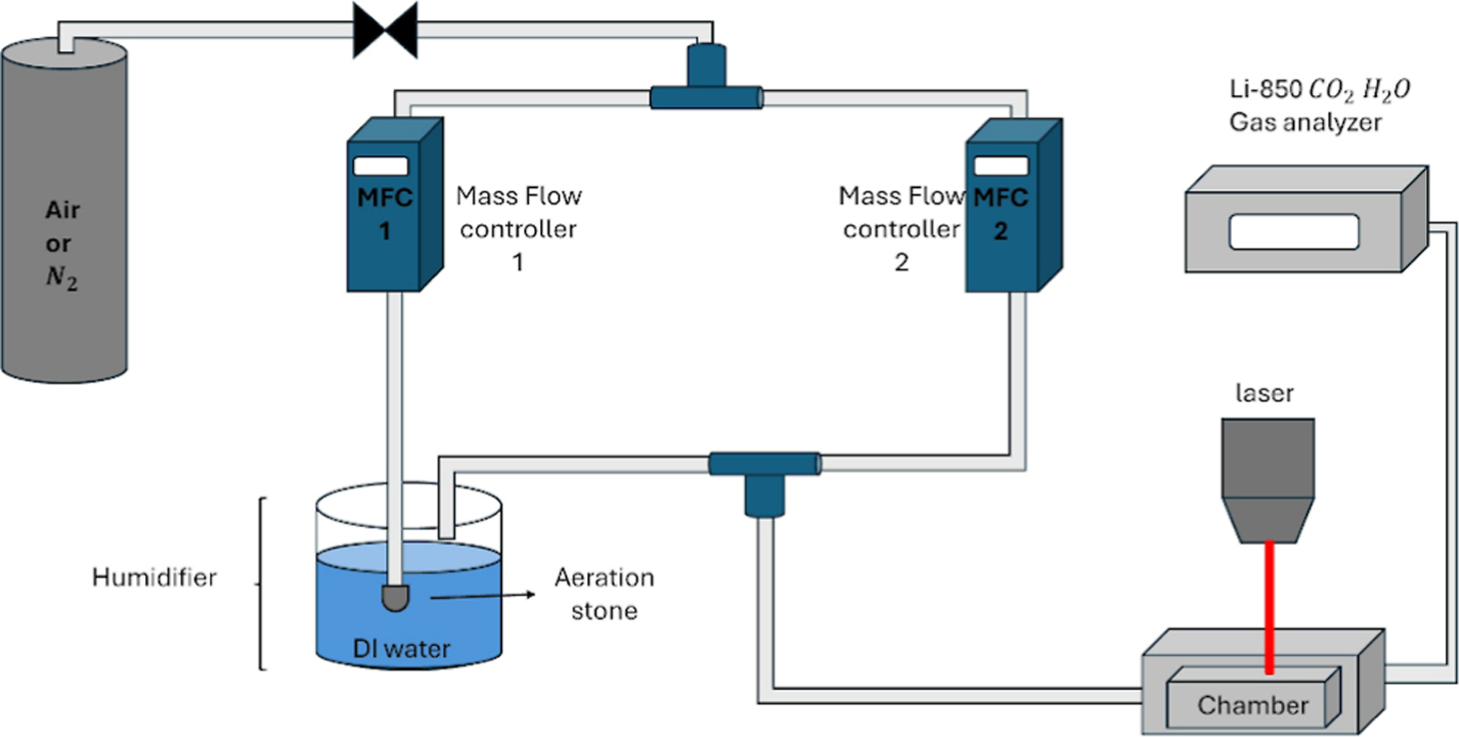
Diagram of the Raman measurement process under dry and wet conditions.

### 2.5 Raman Spectral Analysis

Prior to spectral analysis, all Raman spectra were preprocessed to enhance signal quality and ensure reliable quantification of the anionic species. Initially, spectral smoothing was performed using the Savitzky–Golay method with a second-order polynomial and a 7-point window. This approach effectively reduced high-frequency noise while preserving the integrity of key spectral features such as narrow vibrational peaks associated with bicarbonate and carbonate species. Subsequently, baseline correction was applied to each spectrum to eliminate contributions from background fluorescence and instrumental drift, thereby improving the peak resolution and quantification accuracy. To account for variations in laser power, sample orientation, and acquisition conditions, the spectra were normalized to peaks corresponding to invariant features, serving as an internal standard for spectral normalization. For IRA900, the spectra were normalized to the peak intensity at 1613 cm^–1^, corresponding to the C=C stretching mode of aromatic rings found within the polystyrenic backbone. For AC-KHCO_3_, spectra were normalized to the peak intensity at 1350 cm^–1^, corresponding to the D1-band.^50^ Semiquantification of bicarbonate (HCO_3_^–^) and carbonate (CO_3_^2–^) was performed by integrating the corresponding Raman bands centered at approximately 1017 cm^–1^ and 1065–1068 cm^–1^, respectively, and then normalized to the total spectral area. For the AC-based materials we also examined the hydroxide/water region of the Raman spectra to evaluate any hydroxide species either hydrogen bonded with water (2864 cm^–1^) or free (3706 cm^–1^).^43^ To ensure consistency across all vibrational modes, the OH^–^ band was treated as an individual component within the global fit and its peak area was extracted using the same fitting constraints and optimization parameters. The integrated area of the OH^–^band was subsequently normalized using the same criteria as other bands, to either the total spectral area or the 1350 cm^–1^ D1-band.

To ensure a good spatial representation of the sample, the Raman spectra were averaged over 27 independent scans acquired at different locations across the sample surface. This methodology captures spatial heterogeneity within the sample and provides a statistically robust representation of the bicarbonate-to-carbonate transformation under the experimental conditions.

## 3 Results and Discussion

In the following section, we evaluate two families of moisture-responsive CO_2_ sorbents using open-flow mixed gas sorption and in situ Raman spectroscopy in the presence of dilute CO_2_ (∼400 ppm) and its absence. Here we explore the behavior of a bicarbonate-exchanged macroporous anion exchange resin (IRA900-HCO_3_) and an activated carbon-impregnated with potassium bicarbonate salts (AC-KHCO_3_). The goal of this work is to interrogate the MS mechanism by identifying the dominating reactive species during changes in RH across two different families of materials that exhibit reversible MS.

### 3.1 CO_2_ MS

Calculated cyclic sorbent loadings of CO_2_ (green) and H_2_O (blue), driven by step changes in water activity that oscillate between 20 and 95% RH, are given in Figure 3. The inlet CO_2_ partial pressure was fixed at 40 Pa, and the sorbent temperature was held isothermal at 25 °C. The outlet gas compositions, mass, and heat flows associated with these changes are provided in Figure S1 in the Supporting Information. Both sorbent materials, IRA900-HCO_3_ (solid line) and AC-KHCO_3_ (dotted line), resulted in an increase in water loading from the increase in humidity, as expected. However, the water loading on AC-KHCO_3_ was greater than the polymer resin: 48 ± 2 vs 30 ± 4 mmol/g, where error represents the standard deviation of sorption over three cycles. Further, the rate of higher water loading in the AC-KHCO_3_ did not reach an equilibrium level after 5 h. This higher loading is expected given the higher charge of the impregnated salt (e.g., 5.7 vs 3.0 mmol/g) and the finer micropores comprising activated carbon materials.^51,52^

**Figure 3:**
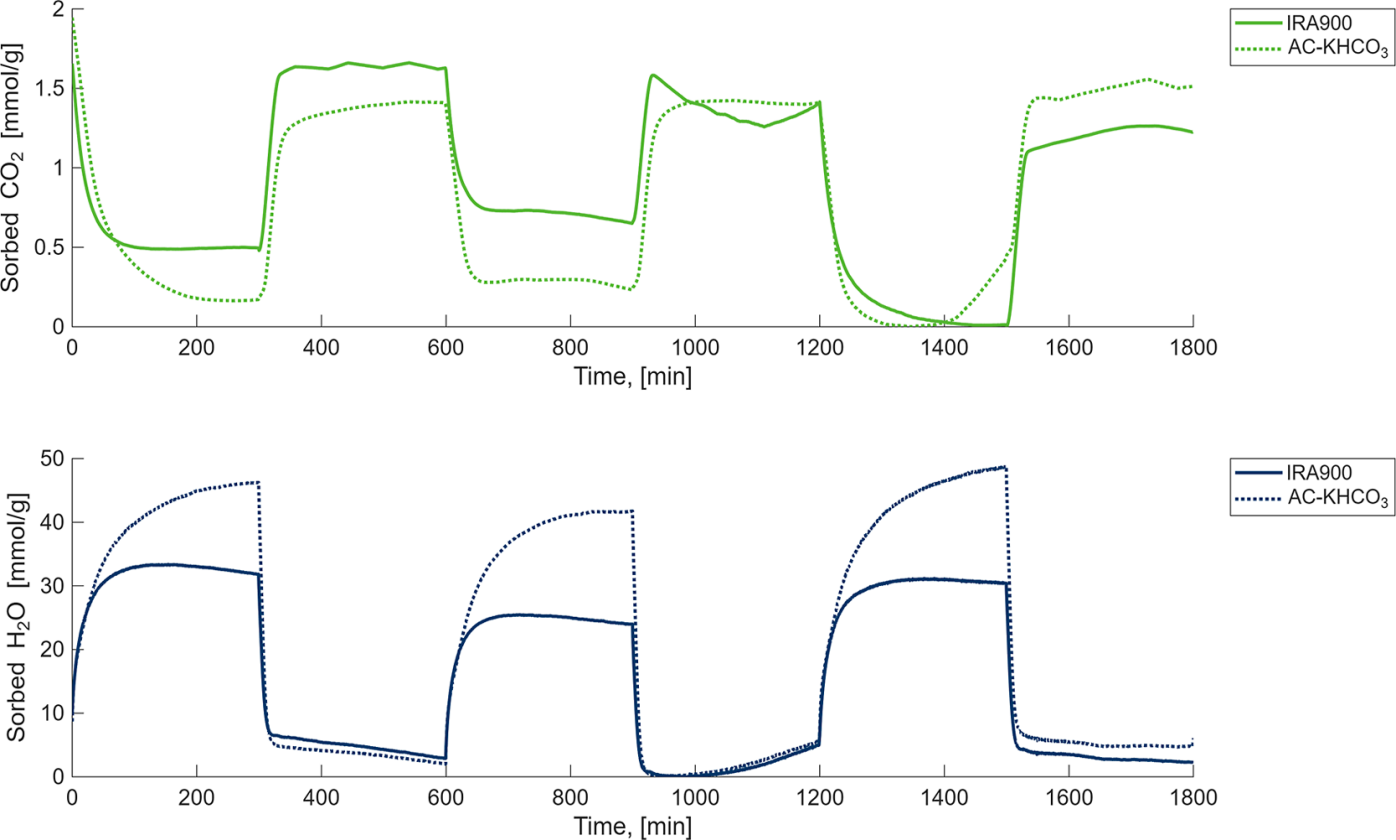
Sorption of CO_2_ (green) and H_2_O (blue) on the IRA900-HCO_3_ (solid) and AC-KHCO_3_ (dashed) sorbents. Experiments were conducted at 25 °C with 40 Pa inlet CO_2_ partial pressure in N_2_. Step increases in water loading are associated with an increase in RH to 95%, decreases in water loading are associated with a decrease in RH to 20%.

As water bound to the sorbent materials, CO_2_ was desorbed and released into the gas phase (see the CO_2_ gas concentration alongside the sorbent mass change in Figure S1). Thus, CO_2_ desorption is counter to H_2_O sorption in the MS process. The calculated CO_2_ loading for both IRA900-HCO_3_ and AC-KHCO_3_ was similar to 1.1 ± 0.2 and 1.4 ± 0.4 mmol/g, respectively. Again, uncertainties represent the average working capacity of the CO_2_ sorption across the three MS cycles. The ratio of H_2_O cycled per unit of CO_2_ separated is thus greater for the IRA900 materials due to the smaller water loadings relative to CO_2_ (11:1 wt/wt vs 13:1 wt/wt H_2_O: CO_2_). Note, the unsmooth plateau of the CO_2_ loadings is an artifact of a drifting CO_2_ gas concentration baseline during those respective cycles, and baseline drift of the H_2_O loading was due to mass balance drift during the experiment resulting in ∼5% error of the H_2_O loading (Figure S1).

As mentioned in Section 2.1, IRA900 was ion-exchanged to 3.0 mmol/g HCO_3_^–^. Upon hydration, bicarbonate is expected to convert to carbonate as CO_2_ desorbs, resulting in a 1:2 ratio of CO_2_ release per pair of charge sites or a theoretical loading of 1.5 mmol/g. At 1.1 mmol/g the sorbent cycles 73% of the theoretical capacity when oscillating between 20 and 95% RH. The bicarbonate impregnation into the AC was 5.7 mmol/g, which leads to a theoretical capacity of 2.9 mmol/g. Only 49% of the theoretical capacity was achieved in the impregnated solid. Thus, in CO_2_-containing atmospheres, we expect to see a partial transition from bicarbonate to carbonate anions in these materials. In the absence of CO_2_ (e.g., in UHP N_2_), the bicarbonate species may also decompose to hydroxide, according to eq 2. All three possible reactive anion species are examined by using Raman.

### 3.2 Surface Enhancement with Nanowires

The morphological characteristics of the sorbents and surface-enhancing nanowires were investigated using scanning electron microscopy (SEM). This technique provides detailed insight into the physical features of both the cryomilled IRA900-HCO_3_ and Ni-coated Ag nanowires (NWs). Figure 4a,b shows SEM micrographs of the Ag and Ni-coated Ag NWs employed as SERS substrates in Raman measurements. The optical properties of the Ni-coated Ag NWs were analyzed by using UV–vis spectroscopy. Figure 4c displays the absorbance spectra of Ag and Ni-coated Ag NWs as a function of wavelength. The blue line represents the absorbance of Ag NWs, revealing two peaks: one at 356 nm, attributed to the out-of-plane quadrupole resonance, and another at 394 nm, corresponding to the out-of-plane dipole resonance of Ag NWs. The red line illustrates the absorbance spectrum of Ni-coated Ag NWs, indicating the suppression of the transverse SPR of Ag NWs at 356 nm, which aligns with nickel deposition on the wire surfaces. A slight red shift is also observed in Ni-coated Ag NWs.^53^ Figure 4d shows TEM images of the synthesized Ni-coated Ag nanowires, which display a uniform, elongated morphology with diameters ranging from approximately 80 to 120 nm and length extending up to several micrometers. Figure 4e presents a high-resolution TEM (HRTEM) image of a nanowire.

**Figure 4:**
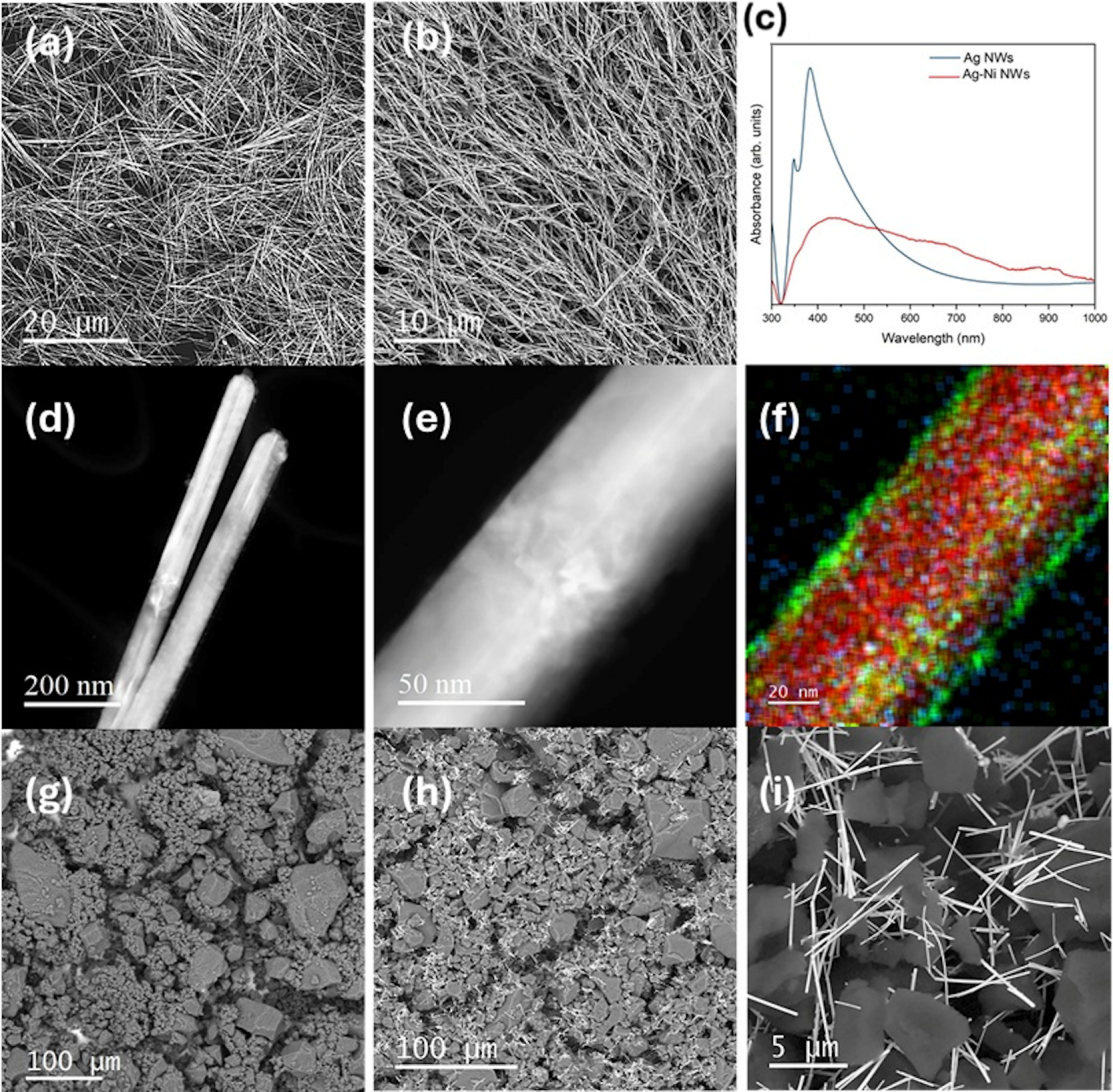
(a) SEM micrograph illustrating the distribution of Ag NWs. (b) SEM micrograph depicting the Ni-coated Ag NWs used in the fabrication of the SERS substrate. (c) UV–vis absorbance spectra of Ag nanowires (blue) and Ni-coated Ag nanowires (red). (d) Low-magnification TEM image showing uniform, elongated Ni-coated Ag nanowires with diameters of ∼80–120 nm. (e) High-resolution TEM (HRTEM) image of the nanowire. (f) EDS elemental mapping of a single nanowire confirming the spatial distribution of silver (red) confined to the core and nickel (green) forming a conformal shell. (g) SEM micrograph of the cryomilled *IRA*900-HCO_3_ sample. (h) SEM micrograph of the *IRA*900-HCO_3_ sample containing nanowires. (i) Higher magnification SEM micrograph of the *IRA*900-HCO_3_ sample with wires, providing a detailed view of the distribution of Ni-coated Ag NWs across the sample.

Energy-dispersive X-ray spectroscopy (EDS) analysis, shown in Figure 4f, illustrates the Ni-coated structure of the nanowires. The image is an RGB composite showing the spatial distribution of Ag (red) and Ni (green) across the nanowire. The Ag mapping is concentrated in the central region, confirming the presence of a metallic silver core. Surrounding the Ag is a distinct green ring, which signifies a coating rich in Ni. Figure 4g,h presents micrographs of the *IRA*900-HCO_3_ cryomilled samples without and with Ni-coated Ag NWs, respectively. Finally, Figure 4i *IRA*900-HCO_3_ shows a micrograph of the sample with Ni-coated Ag NWs, highlighting the distribution of these NWs within the sample.

Figure 5a shows a scanning electron microscopy (SEM) image of the IRA900 resin prior to cryomilling. The SEM image in Figure 5b reveals a porous surface. A higher magnification image in Figure 5c highlights the presence of macropores distributed across the surface. The pore size distribution of the IRA900 sample is presented in Figure 5d as a histogram of pore diameters (nm). A total of 498 individual pores were measured to construct the histogram, providing a statistically robust representation of the sample’s morphology. The height of each bar represents the number of pores within the corresponding size range. The distribution exhibits a narrow peak with an average pore diameter of approximately 80 nm, indicative of a relatively uniform porosity. The macro-pore size distribution of IRA900–HCO_3_ plays a critical role in dictating the mass transfer of CO_2_ and H_2_O to and from the surface of the particle. Finer pore sizes, approaching that of the free volume fraction between the polymer strands of the resin, can only be achieved through alternative methods like N_2_ adsorption with a pore analysis, see for example,^26,27^ which detail the 1–10 nm pore domains using both nonlinear density functional theory (NLDFT) and Barrett–Joyner–Halenda BJH analyses.

**Figure 5:**
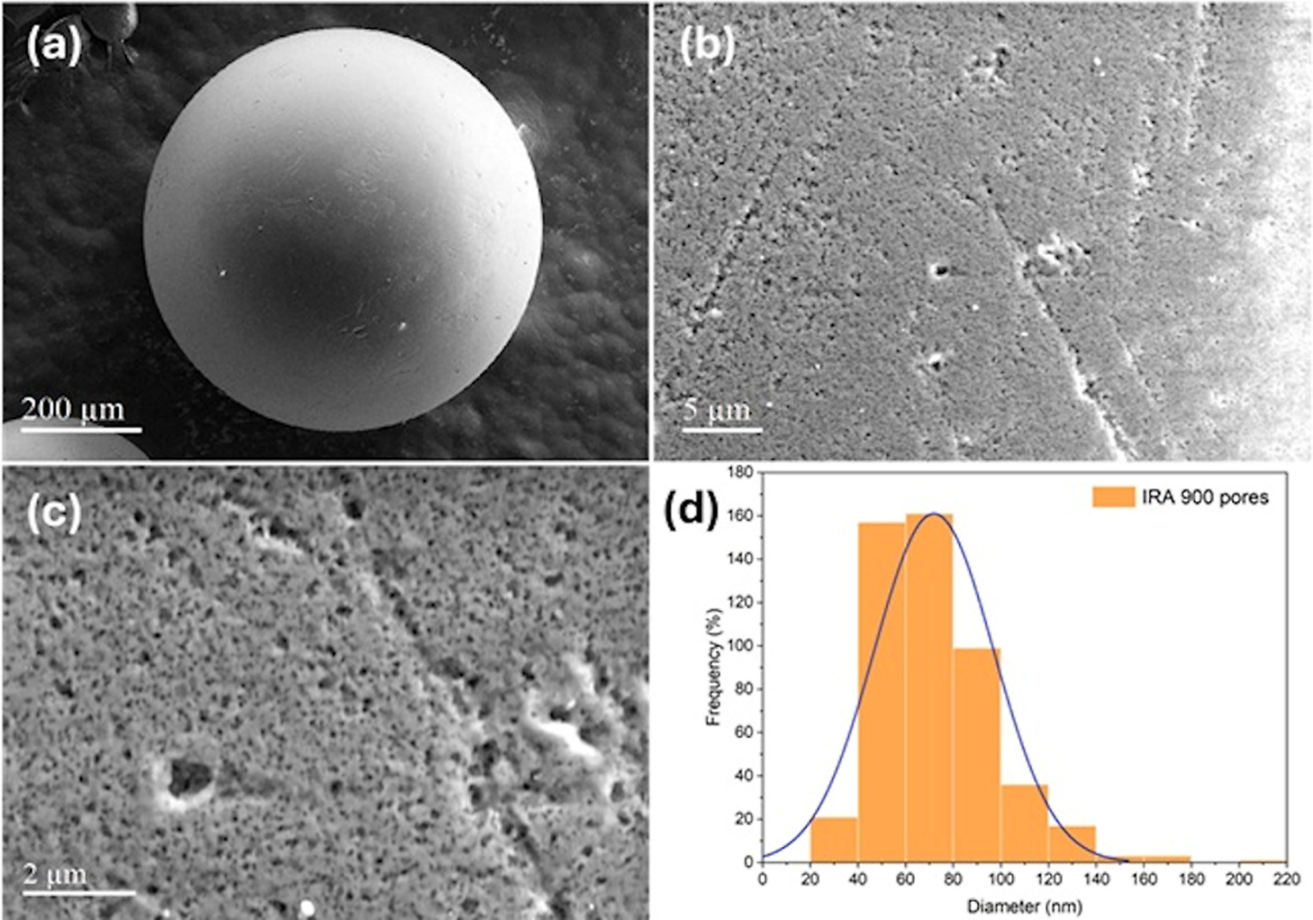
(a) SEM image of the IRA900-HCO_3_ sample prior to the cryomilling process. (b) SEM image displaying the surface morphology of the IRA900-HCO_3_ sample after cryomilling, where the presence of macropores is evident. (c) High-magnification SEM image of the cryomilled IRA900-HCO_3_ sample, highlighting the variation in pore sizes across the surface. (d) Histogram of the pore size distribution obtained from SEM image analysis of cryomilled IRA900-HCO_3_. The average pore diameter was determined to be approximately 80 nm with standard deviations of <10%.

The SERS effect was evaluated on both the control IRA900-Cl and the reactive IRA900-HCO_3_ samples with and without Ni-coated Ag NWs under dry conditions. Figure 6a shows Raman measurements of *IRA*900-HCO_3_ with (blue) and without (red) Ni-coated Ag NWs, and IRA900-Cl with Ni-coated Ag NWs (black) as a function of the Raman shift under dry N_2_ conditions.

**Figure 6:**
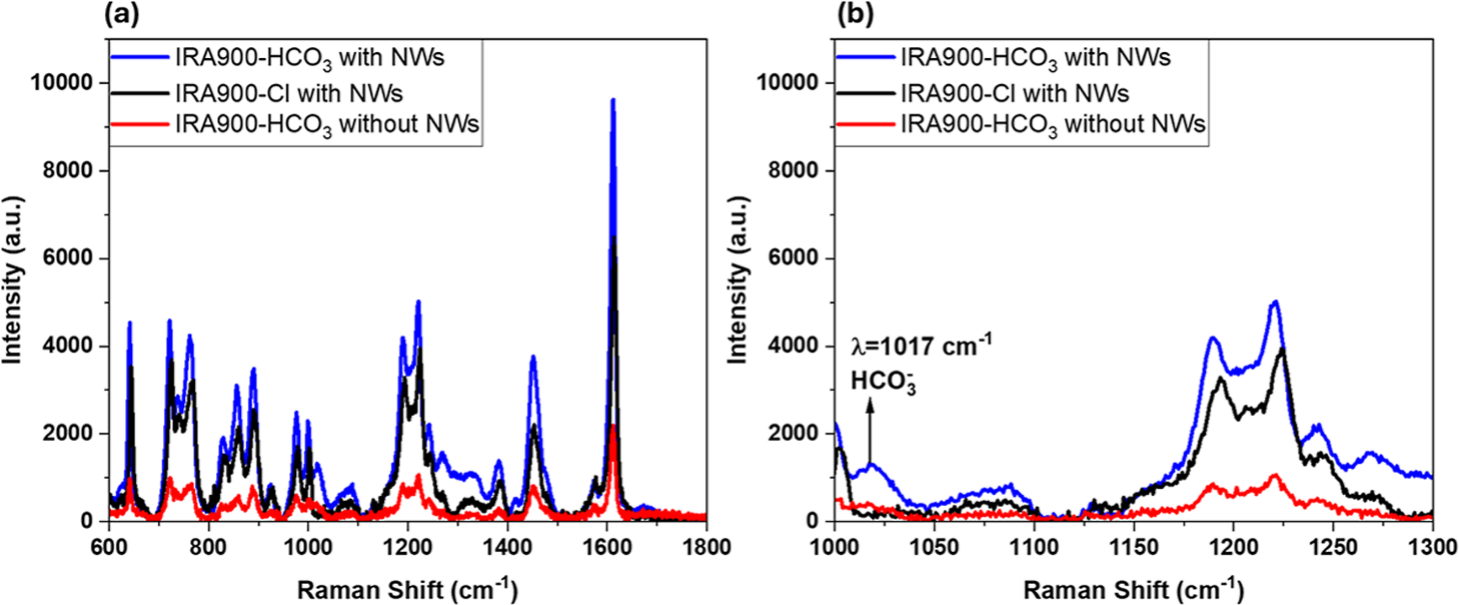
(a) Raman spectra of *IRA*900-HCO_3_ with Ni-coated Ag NWs (blue line), the control IRA900-Cl with Ni-coated Ag NWs (black line), and *IRA*900-HCO_3_ without Ni-coated Ag NW (red line) samples as a function of wavelength shift under dry N_2_ conditions. (b) Spectrum of the *IRA*900-HCO_3_ sample shows a distinct peak at 1017 cm^–1^, corresponding to the characteristic band of HCO_3_^–^.

The Raman spectrum of the *IRA*900-HCO_3_ samples with NWs exhibits a significant increase in Raman intensity, referred to as the enhancement factor (EF), compared to the spectrum of the *IRA*900-HCO_3_ without. This enhancement arises from the SERS effect generated by the interaction between the Ni-coated Ag NWs, and the red laser used during the Raman measurements. The spectrum of the *IRA*900-HCO_3_ sample shows a distinct 1017 cm^–1^ peak, characteristic of HCO_3_^–^ under dry conditions, which is absent in IRA900-Cl (Figure 6b).^54,55^ We note that Raman measurements in the 2800–3800 cm^–1^ region, corresponding to O–H stretching modes, were not possible for IRA900–HCO_3_ with red laser excitation (633–785 nm), as strong absorption by the polymer matrix caused localized heating and irreversible degradation. Thus, the inability to probe this region arises from the intrinsic photothermal sensitivity of IRA900–HCO_3_, not the absence of Raman-active modes.

To calculate the EF, eq 4 was used

4 $$EF=\frac{I_{SERS}\cdot n_{Raman}}{I_{Raman}\cdot n_{SERS}}$$

where EF is SERS enhancement factor, *I*_SERS_ (Figure S3) is the Raman intensity using SERS, *I*_Raman_ is the Raman intensity without using SERS, *n*_SERS_ represents the number of molecules in contact with the SERS substrate within the probed area, while *n*_Raman_ denotes the number of molecules present within the laser’s excitation volume during standard Raman scattering measurements.^56–58^ The EF was calculated based on the bicarbonate vibrational band centered at 1017 cm^–1^ under dry nitrogen (Dry-N_2_) conditions. The measured intensity of this peak was 1200 arbitrary units (au) in the presence of the SERS substrate, compared to 200 au in its absence. After performing the calculations, an EF of 3.0 *×* 10^9^ was obtained. All EF calculations are detailed in eqs S2–S10.

### 3.3 CO_2_ Reactivity in Ion-Exchange Resin

To assess anion speciation changes from CO_2_ (de)­sorption with IRA900–HCO_3_ (eqs 1–3), Raman spectra were collected before and after a step increase in RH. Figure 7a shows control data for IRA900–Cl under dry and humid air (PCO_2_–430 ppm) over 3 h. No time-dependent spectral changes are observed, and characteristic bicarbonate (HCO_3_^–^, 1017 cm^–1^) and carbonate (CO_3_^2–^, 1065 cm^–1^) bands are absent, confirming that the chloride system does not respond to variations in humidity.

**Figure 7:**
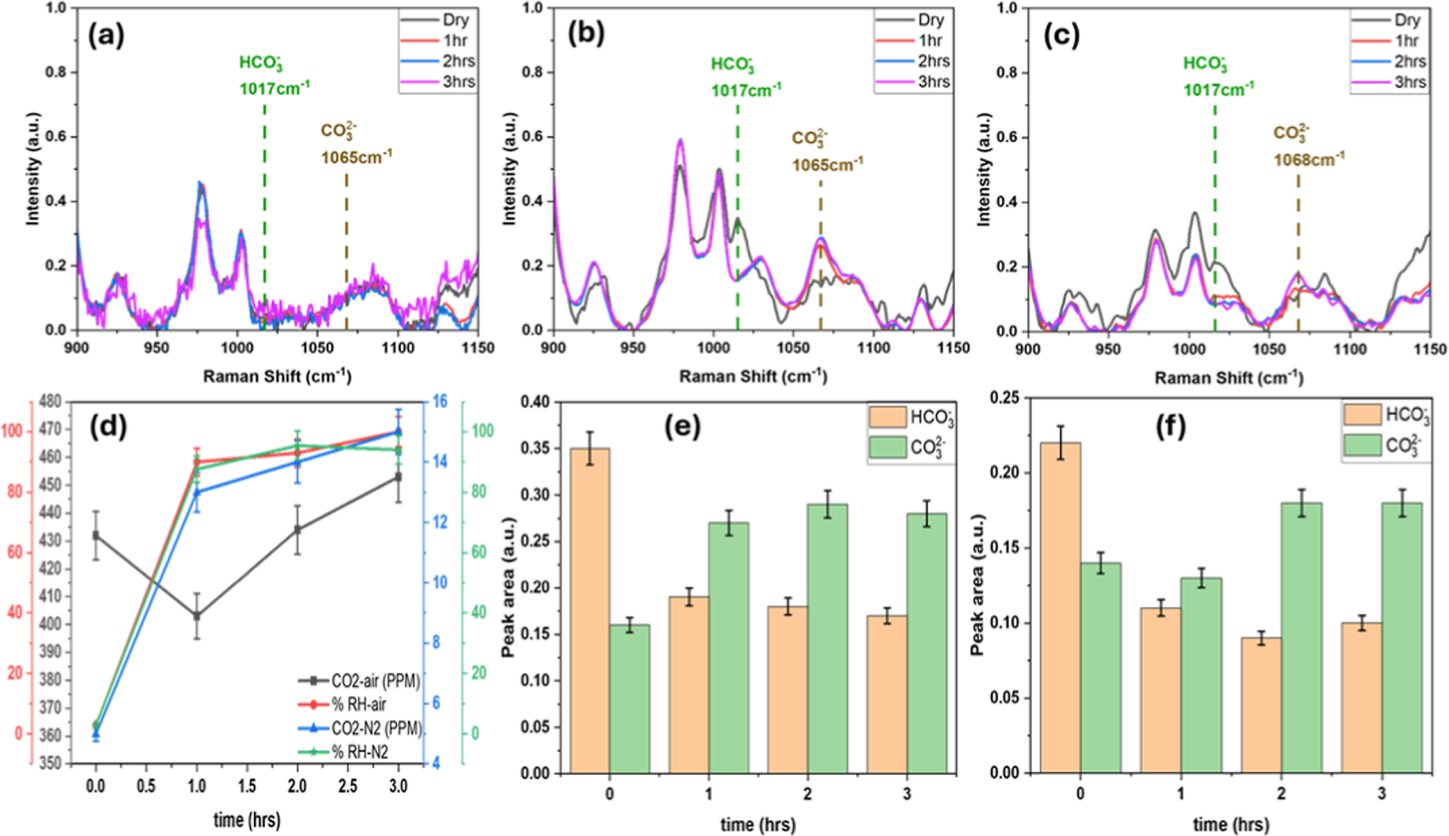
Time-evolved Raman spectra of (a) IRA900-Cl in air, (b) *IRA*900-HCO_3_ in air, and (c) *IRA*900-HCO_3_ in N_2_ during a step change from low to high humidity. (d) Time series measurements of sample chamber gas composition as CO_2_ (black) and %RH (red) in air and CO_2_ (blue) and %RH in N_2_ (green). (e,f) Bar graphs of integrated Raman band areas corresponding to HCO_3_^–^ and CO_3_^2–^ species under air (e) and N_2_ (f). Results indicate a progressive conversion of bicarbonate to carbonate under humid conditions, particularly in an inert N_2_ atmosphere.

Figure 7b,c displays the time evolution of IRA900-HCO_3_ Raman spectra in air (b) and N_2_ (c) while undergoing a step increase in humidity from 2.8 to 95% RH. Figure 7d shows the correlating change in gas concentration over time in the sample chamber from the initial dry state to the humidified state, with CO_2_ reported in parts per million, ppm (black in air, blue in N_2_) and % RH (red in air, green in N_2_). Under dry air (420 ppm of CO_2_), the Raman spectrum exhibits a distinct HCO_3_^–^ band at 1017 cm^–1^ (35% integrated area relative to the 1615 cm^–1^ peak) and a smaller CO_3_^2–^ band at 1065 cm^–1^ (16%). Under dry N_2_ (<10 ppm of CO_2_), similar features are observed, though with lower relative intensities (22% for HCO_3_^–^ and 14% for CO_3_^2–^), yielding a lower HCO_3_^–^/CO_3_^2–^ ratio, 1.6 vs 2.2 in air. In both conditions, an increase in RH corresponds to an increase in the CO_3_^2–^ area and corresponding decreases in the HCO_3_^–^ area, supporting the predicted MS mechanism described by (eq 3). Error bars in Figure 7e,f correspond to the standard deviation of these measurements that have been added to reflect the spatial reproducibility of the data.

Humidification in the presence of CO_2_ (Figure 7b,e) produces a 50% decline in the HCO_3_^–^ peak area with the fastest change within the first hour. This rate aligns with sorption kinetics observed in RH cycling experiments in 400 ppm of CO_2_ (Figure 3). Concurrently, the intensity of the CO_3_^2–^ signal initially rises by 88% before declining to 75% of its starting value. Stoichiometric charge balance predicts that for every CO_2_ molecule released, two HCO_3_^–^ are consumed per CO_3_^2–^ formed (eq 3), but the Raman data deviate from this expectation. One possible explanation is a hydration-induced shift of the 1017 cm^–1^ band, with a new feature emerging at 1020–1050 cm^–1^. This could reflect altered hydrogen bonding or an unidentified phase that convolutes the HCO_3_^–^ signal. The persistence of a residual HCO_3_^–^ band is consistent with the MS sorption data, where the capacity oscillates by only 1.1 mmol g^–1^ out of 3.0 mmol g^–1^ total exchange capacity, indicating incomplete participation of HCO_3_^–^ in the swing.

Hydration in N_2_, Figure 7c,f, causes a stronger decrease in the HCO_3_^–^ peak (74%) with a smaller increase in CO_3_^2–^ (∼29%). This pattern better matches the stoichiometric expectation of the MS but suggests an additional process at a low PCO_2_. Specifically, hydroxide formation may emerge under these conditions, explaining the overall (bi)­carbonate signal suppression and muted carbonate response compared to air. We note again that detecting the hydroxide phase directly in the O–H stretching region (2800–3800 cm^–1^) was not possible for IRA900–HCO_3_ using red excitation (633–785 nm) due to photothermal degradation of the polymer.

### 3.4 CO_2_ Ion Reactivity in Bicarbonate-Impregnated Porous Carbon

Like the IRA900 samples, reactive ion speciation was examined in *AC*-KHCO_3_, while undergoing the same step increase in humidity. Figure 8a shows a morphological overview of the activated carbon material impregnated with KHCO_3_. At higher magnification (Figure 8b), the carbon matrix displays a well-developed porous network formed by chemical activation. Further magnification (Figure 8c) reveals a homogeneous distribution of nanoscale pores, indicating uniform etching across the carbon walls.

**Figure 8:**
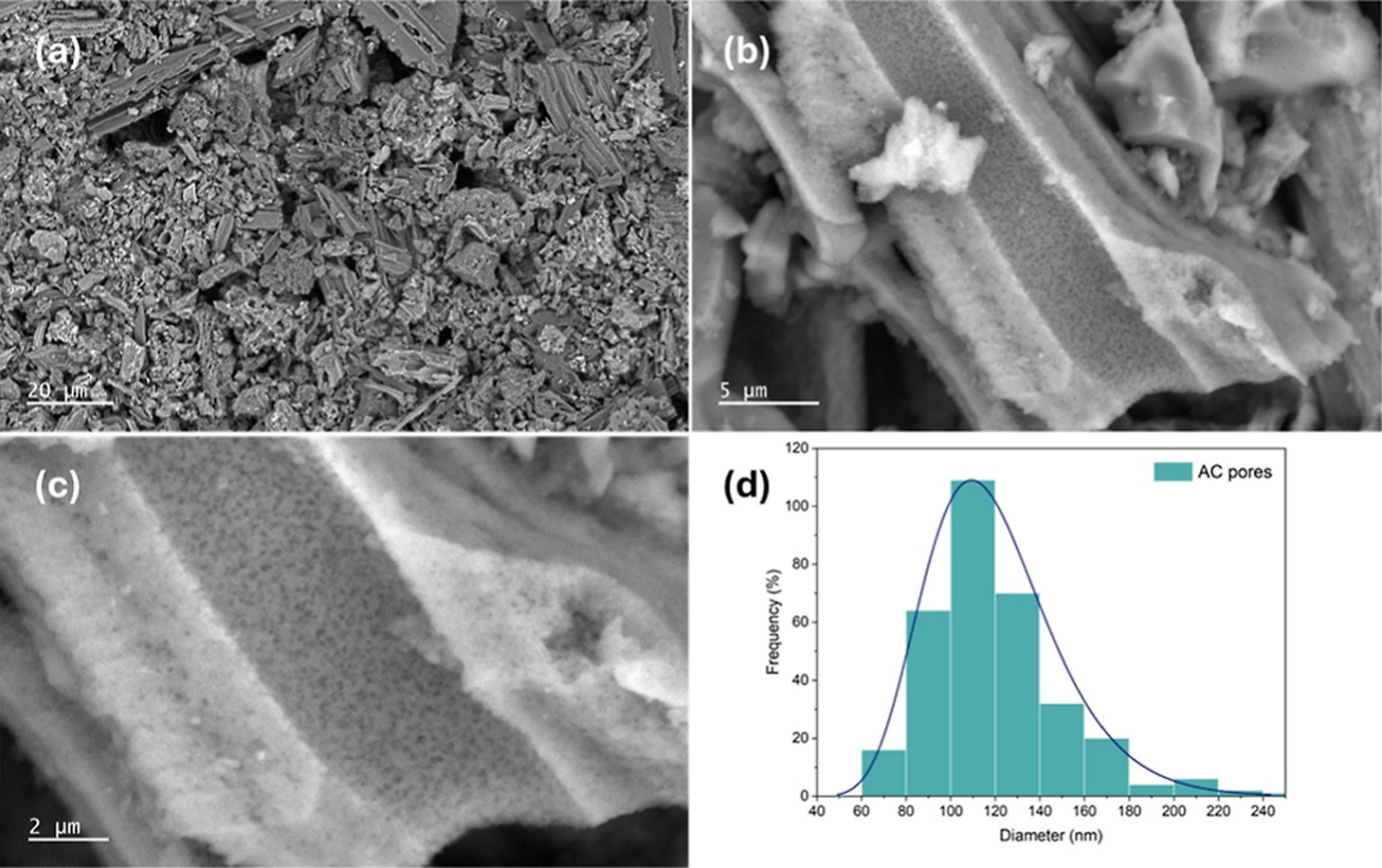
(a) Overview of the surface morphology of the activated carbon impregnated with KHCO_3_. (b) Magnified view revealing the porous texture formed on the activated carbon walls. (c) Close-up image highlighting a uniform distribution of nanoscale pores along the walls. (d) Pore size distribution histogram derived from SEM image analysis, showing an average diameter of approximately 110 nm with standard deviations of <10%.

To quantitatively characterize this morphology, a pore size distribution analysis was conducted from the SEM images (Figure 8d). A total of 420 individual pores were measured to construct the histogram, where the *y*-axis represents the count of pores within each size range. The distribution is narrowly centered around 110 nm, confirming its macroporous character. Such pores are particularly advantages for gas adsorption because they enable rapid transport of gas species to/from the sorbent surface.

Comparison with IRA-900 highlights notable textural similarities despite differences in the chemical composition. Both materials exhibit pore sizes in the macroporous regime (50–200 nm) and broad, unimodal distributions, suggesting well-connected channel networks that facilitate the mass transport of the two sorbates to the sorbent surface. These macroporous structures therefore help explain the comparable kinetics observed in the humidity-driven bicarbonate–carbonate conversion shown in Figure 3. It should be noted, however, that features below 10 nm—known to dominate the surface area of activated carbon—were not resolved here, as this lies beyond the resolution of the SEM. Details of the microporous texture of salt-impregnated activated carbon sorbents are provided in Zheng et al.^18^

Time-resolved Raman spectra of AC–KHCO_3_ under humidified air and N_2_ environments are shown in Figure 9. Unlike the IRA900 scans, a wider spectral window was collected, extending to 3800 cm^–1^ to capture both the HCO_3_^–^/CO_3_^2–^ region and the O–H stretching modes. All spectra were baseline-corrected and normalized to the D band (∼1350 cm^–1^) to ensure consistency in comparisons. In air (Figure 9a), the O–H stretching region shows no distinct peaks in the dry state (black). After 1 h (red), the signal intensity increases to 2.1 (arbitrary units) of the normalized peak intensity and after 2 h (blue) by 4.0 (arbitrary units). Peak deconvolution in this region (Figure S4 and Table S1) identifies seven Gaussian components (2879–3622 cm^–1^), which correspond to hydrogen-bonded water species, hydrogen bonded OH^–^ (2900 cm^–1^), and free OH^–^ ions (3500 cm^–1^), consistent with literature assignments.^43^

**Figure 9:**
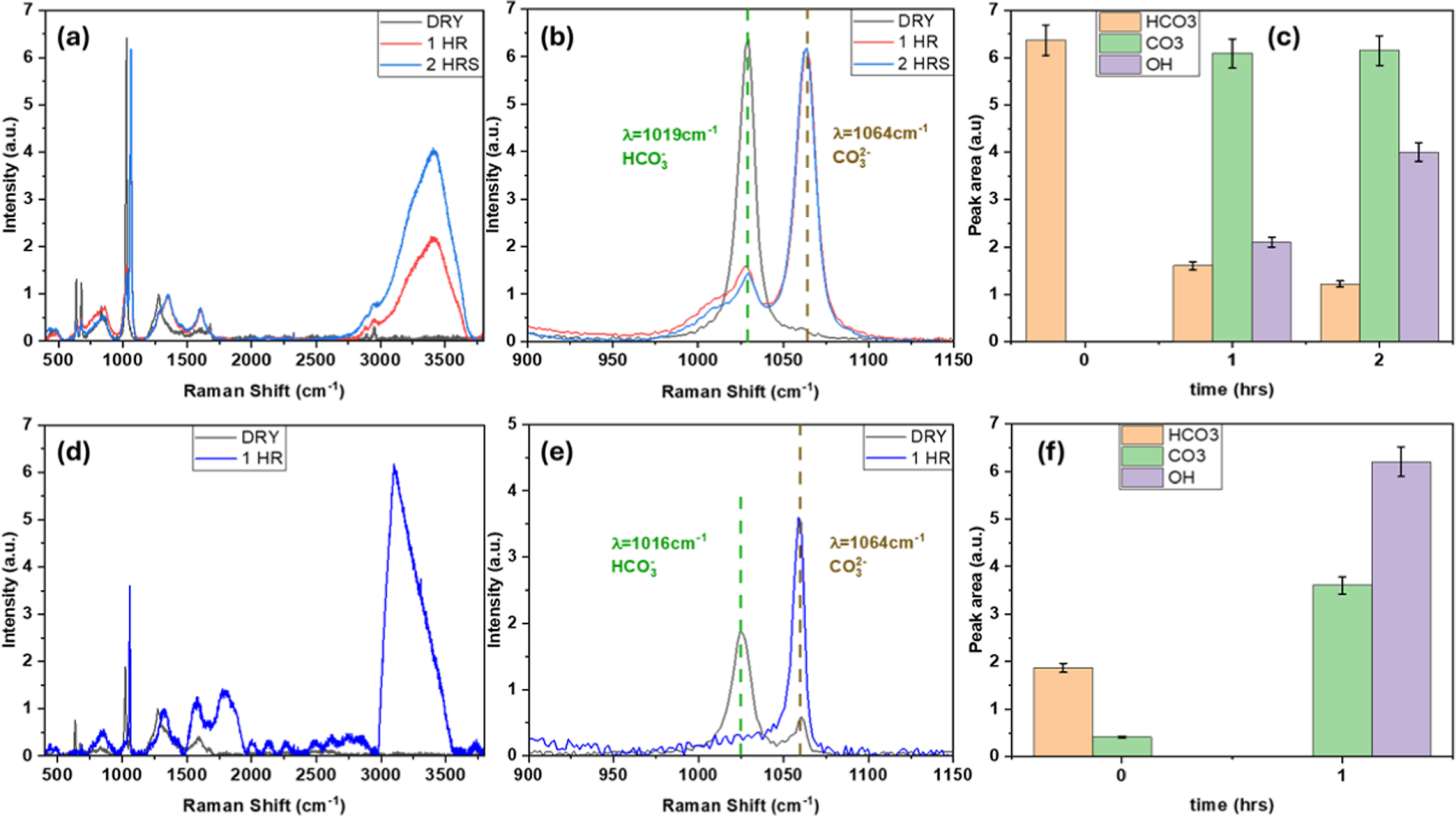
Time-dependent Raman analysis of AC–KHCO_3_. (a–c) show spectra under humid air; (d–f) under humid N_2_. (a) Full-range Raman spectra in dry (black) and after 1 h (red) and 2 h (blue) of humid air exposure. (b) Zoomed 900–1150 cm^–1^ region showing HCO_3_^–^ decline (1019 cm^–1^) and CO_3_^2–^ rise (1064 cm^–1^). (c) Normalized peak areas of HCO_3_^–^, CO_3_^2–^, and OH^–^. (d) Full-range spectra under dry (black) and 1 h humid N_2_ (blue). (e) Zoomed region showing HCO_3_^–^ loss (1016 cm^–1^) and CO_3_^2–^ increase (1060 cm^–1^). (f) Normalized peak areas reveal rapid bicarbonate-to-carbonate conversion under humid N_2_. Error bars represent the standard deviation from spatially averaged spectra.

The carbonate region (900–1200 cm^–1^, Figure 9b) also evolves strongly with humidity. Under dry air, only a strong HCO_3_^–^ band is observed, comprising 6.4 of the spectral intensity. Gaussian deconvolution of this region is presented in Figure S5 with peak positions and heights quantified in Table S2. After 1 h in air, this bicarbonate peak diminishes by ∼75% while a strong CO_3_^2–^ band emerges at 1064 cm^–1^ accompanied by a small OH^–^ signal. After 2 h, bicarbonate declines slightly further, carbonate intensity plateaus, and the OH^–^ contribution increases from ∼0.4 to ∼0.6 of the normalized peak area. Figure 9c summarizes these changes, showing a first sharp then slow decline in bicarbonate concurrent with carbonate formation and gradual hydroxide growth. This behavior mirrors CO_2_ desorption in AC-KHCO_3_ sorption experiments at 400 ppm of CO_2_ (Figure 3), providing mechanistic evidence for the moisture-swing reactions described by eqs 1–3.

The same experiment in N_2_ (Figure 9d,e) shows important differences. In the dry state, both HCO_3_^–^ and CO_3_^2–^ are already present in a ∼4:1 ratio, consistent with partial bicarbonate decomposition in the absence of CO_2_ (eq 2). After 1 h of humidified N_2_ exposure, the bicarbonate peak disappears entirely, replaced by a much stronger CO_3_^2–^ signal and an emerging OH^–^ band that is deconvoluted in Figure S6 with peak assignment in Table S3. Deconvolution of the carbonate region identifies five components (1016–1060 cm^–1^; Figure S7 and Table S4), with initial relative intensities of 27.3% for HCO_3_^–^ and 6.9% for CO_3_^2–^. Upon hydration, the sharp growth of carbonate to ∼43% and concurrent OH^–^ formation confirm a strong driving force toward CO_2_ desorption when PCO_2_ concentration is very low.

A direct comparison of the two environments underscores these differences. In both cases, bicarbonate decreases while carbonate and hydroxide grow, but the transformation is faster in N_2_, with bicarbonate fully depleted in under 1 h. The presence of carbonate even in the initial dry N_2_ spectrum, coupled with depletion of the carbonate signal, indicates that low PCO_2_ accelerates conversion by destabilizing bicarbonate and favoring CO_2_ release. By contrast, atmospheric CO_2_ (∼430 ppm) in air stabilizes bicarbonate, sustaining a higher initial HCO_3_^–^ intensity and moderating the conversion rate.

Taken together, the gas sorption and in situ Raman experiments on IRA900 and AC–KHCO_3_ provide direct evidence of the MS mechanism described in eqs 1–3, while also clarifying how hydration and PCO_2_ govern anion speciation in distinct host materials. For IRA900, bicarbonate dominates under dry air (∼420 ppm of CO_2_) but diminishes by ∼50% within the first hour of humidification, with carbonate intensity rising more strongly than stoichiometry predicts. The excess carbonate signal likely reflects the enhanced Raman scattering efficiency of CO_3_^2–^ rather than true ion ratios, underscoring the challenge of extracting quantitative speciation from Raman peak areas. A residual bicarbonate band persists even under extended hydration, consistent with sorption data showing incomplete cycling of exchange sites. Under N_2_ (<10 ppm of CO_2_), bicarbonate is less stable, declining by ∼74% with only a modest carbonate increase (∼29%), suggesting hydroxide formation competes at low PCO_2_. Interpretation of hydroxide speciation, however, is constrained by the inability to probe the O–H stretching region in the polymer due to photothermal degradation.

In contrast, the AC–KHCO_3_ system could be probed across a wider spectral window (to 3800 cm^–1^), enabling simultaneous observation of carbonate, bicarbonate, and O–H stretching features. Under humid air, bicarbonate declines by ∼75% after 1 h, accompanied by a strong carbonate peak and a small hydroxide signal. After 2 h, carbonate intensity plateaus while hydroxide grows, consistent with the role of OH^–^ in eq 2. In N_2_, the driving force for CO_2_ desorption is more pronounced: carbonate is already present in the dry state, bicarbonate is fully depleted within 1 h, and hydroxide contributions increase. This behavior reflects Le Chatelier’s principle, where the absence of gas-phase CO_2_ destabilizes bicarbonate and accelerates conversion to carbonate and hydroxide. Unlike polymeric IRA900, the carbon matrix allows the direct detection of hydroxide vibrational modes, providing stronger mechanistic evidence that OH^–^ participates in the hydration-dependent equilibrium. This has important implications for the expected working capacity from these sorbents in real sorption processes where PCO_2_ can be driven down with a water vapor sweep to enhance CO_2_ release.

Overall, both materials confirm the hydrolysis reactivity of carbonate as the core driver of the MS process, but their structural and spectroscopic properties highlight complementary aspects of the mechanism. IRA900 demonstrates how polymer-supported bicarbonate persists under ambient CO_2_, though stoichiometric interpretation is limited by spectral bias and inaccessible O–H modes. AC–KHCO_3_, by contrast, reveals the carbonate–bicarbonate–hydroxide interplay across the vibrational spectrum and highlights the stronger desorption-driving force under low PCO_2_. Together, these findings validate the proposed equilibrium framework and emphasize the importance of both hydration and the CO_2_ partial pressure in controlling sorbent performance while also pointing to methodological limitations that must be addressed in future studies.

## 4 Conclusion

This study demonstrates the effective use of in situ SERS to resolve ion speciation dynamics in MS sorbents under controlled humidities and gas environments. By leveraging Ni-coated Ag nanowire-based SERS substrates, we achieved substantial enhancement in vibrational signal detection, enabling real-time monitoring of carbonate, bicarbonate, and hydroxide species within anion-exchange resins (*IRA*900-HCO_3_) and KHCO_3_^–^-impregnated activated carbon (*AC*-KHCO_3_). The observed reversible transitions between bicarbonate and carbonate in response to water activity shifts provide direct experimental validation of the humidity-driven equilibrium central to the MS mechanism. Importantly, this work establishes SERS as a practical, nondestructive characterization tool capable of operando analysis of reactive interfaces in DAC sorbents. The methodology enables molecular-level feedback on sorbent chemistry under operating conditions, supporting the accelerated development of MS materials with improved CO_2_ capture efficiency, regeneration kinetics, and operational stability. This platform opens avenues for quality control and material screening in scalable carbon capture technologies aligned with climate mitigation goals.

## Supplementary Material



## Notes

Detailed calculations of the EF for Ni-coated Ag NWs, highlighting their suitability for fabricating SERS substrates in Raman measurements; TEM and SEM analyses used to examine the structural and surface morphology of the synthesized materials; Raman spectra of the *IRA*900-HCO_3_ and *AC*-KHCO_3_ membranes preprocessed through baseline correction and normalization; and peak deconvolution using Gaussian fitting applied to resolve overlapping bands, enabling identification of specific vibrational modes and chemical interactions under different environmental conditions (PDF)

## Abbreviations

CO_2_: carbon dioxide
MS: moisture swing
SERS: surface-enhanced Raman spectroscopy
LSPR: localized surface plasmon resonance
SPPs: surface plasmon polaritons
Ni: nickel
Ag: silver
Au: gold
Ni-coated Ag NWs: Ni-coated Ag nanowires
N_2_: nitrogen gas
PPT: part per thousand
PPM: parts per million
MFC 1: mass flow controller 1
MFC 2: mass flow controller 2
*I*_SERS_: Raman intensity using SERS
*I*_Raman_: Raman intensity without using SERS
*n*_SERS_: number of molecules adsorbed on the SERS substrate within the probed area
*n*_Raman_: number of molecules present within the laser’s excitation volume during standard Raman scattering measurements
a.u.: arbitrary units
%RH: percentage of Relative Humidity
DAA: donor–acceptor–acceptor
DDAA: donor–donor–acceptor–acceptor
DA: donor–acceptor
