# Supplementary material for: Surface-Enhanced Raman Detection of the CO2 Moisture Swing
Source: ACS Appl Eng Mater. 2025 Nov 5;3(11):4050–62. doi: 10.1021/acsaenm.5c00716 (PMC12671539; doi:10.1021/acsaenm.5c00716)
Supplement: Supplementary file 1 [file em5c00716_si_001.pdf]

Supporting Information

Surface Enhanced Raman Detection of the CO_2 Moisture Swing

Javier Mendez Lozoya[1,3], *Estrella Solis Mata*[2], *J. Jesus Velazquez Salazar*[1,2], *Alondra Hernandez Cedillo*[1,2], *Miguel Jose Yacaman*[1,2], *and Jennifer L. Wade*[4*]

[1] Department of Applied Physics and Materials Science, Northern Arizona University, Flagstaff, Arizona, 86011, USA

[2] Center for Materials Interfaces in Research and Applications, Northern Arizona University, Flagstaff, Arizona 86011, USA

[3] Instituto Tecnológico de San Luis Potosí, Tecnológico Nacional de México, Av. Tecnológico s/n, Soledad de Graciano Sánchez, San Luis Potosí 784 n n37, México

[4] Jennifer.Wade@nau.edu; Steve Sanghi College of Engineering, Department of Mechanical Engineering, Northern Arizona University, Flagstaff Arizona 86011

Supporting Information

The open flow, 25 °C CO_2 moisture swing experiments for the two sorbent samples are shown in **Figure S1**. Further, a control experiment with the non-reaction IRA900-Cl sorbent is shown in **Figure S2** for comparison. In all runs, water vapor was oscillated in the input between 20-95% RH at the location of the sample. This oscillation is apparent in both the outlet water vapor concentration (blue) and sample mass (black). CO_2 was held constant in the inlet and the spikes in CO_2 concentration seen in Figure S1 correspond to desorption when moisture increases and chemisorption when moisture decreases. In contrast, IRA900-Cl shows no CO_2 perturbations beyond the dilution effect of added water vapor. Similar non-reactive behavior was observed for an AC control without impregnated salt. The slight baseline shift in CO_2 concentration arises from dilution with water vapor: as water vapor increases, CO_2 decreases by ~5 ppm, and the reverse occurs as water vapor decreases. We did not apply a dilution correction since baseline integration was taken over the final 60 minutes of a 5-hour hold, when CO_2 and H_2O concentrations were relatively steady. This effect is evident in both the reactive samples (Figure S1) and the IRA-900-Cl control (Figure S2).

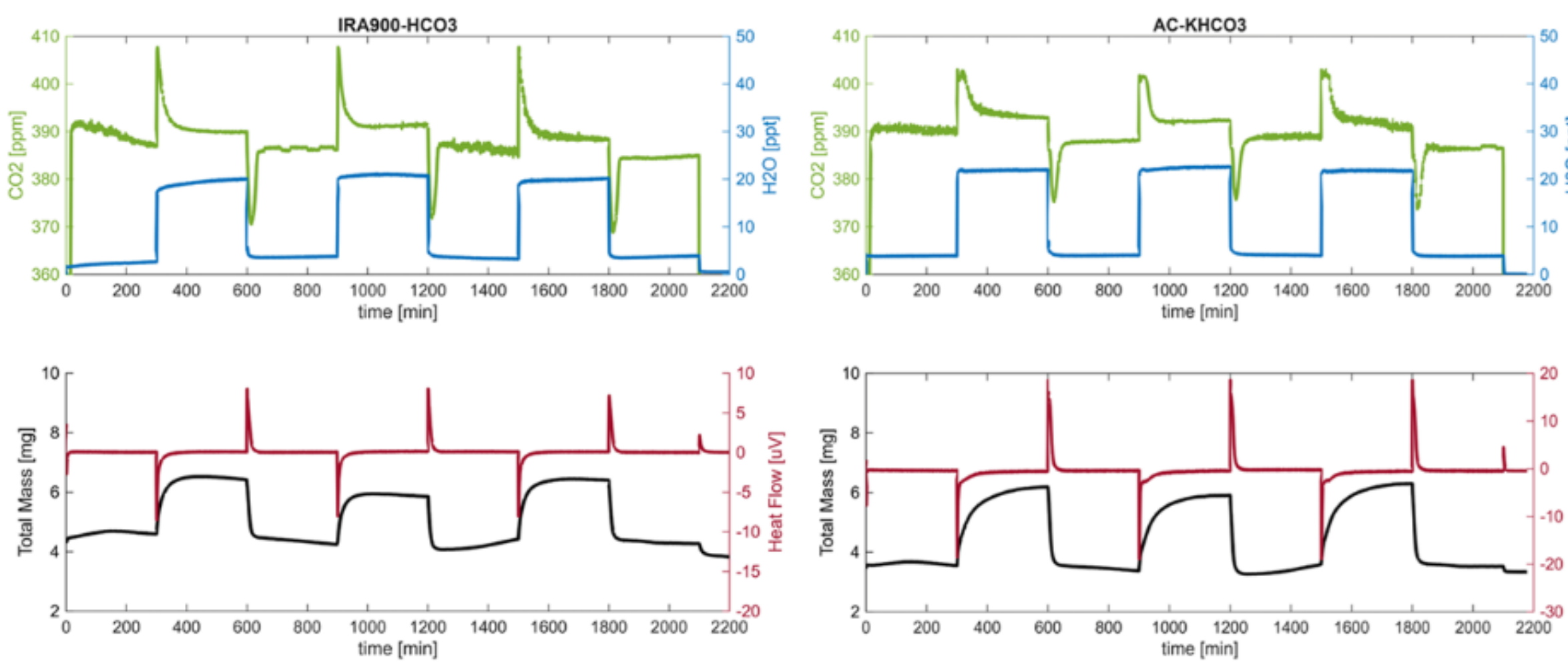

Figure S1 *Measurements of the CO_2 and H_2O dual sorption experiments for IRA900-HCO3 (left) and AC-HCO3 (right). The gas analyzer detected CO_2 (green) and H_2O (blue) concentrations evolved from the outlet of the TGA/DSC instrumentation. An upward spike in CO2 corresponds to CO2 desorption from the sample The changing mass (black) is due to both water and CO2 sorption, and the corresponding net heat flow (red) from the net sorbent changes is dominated by water sorption, where a downward spike corresponds to an exothermic event.*

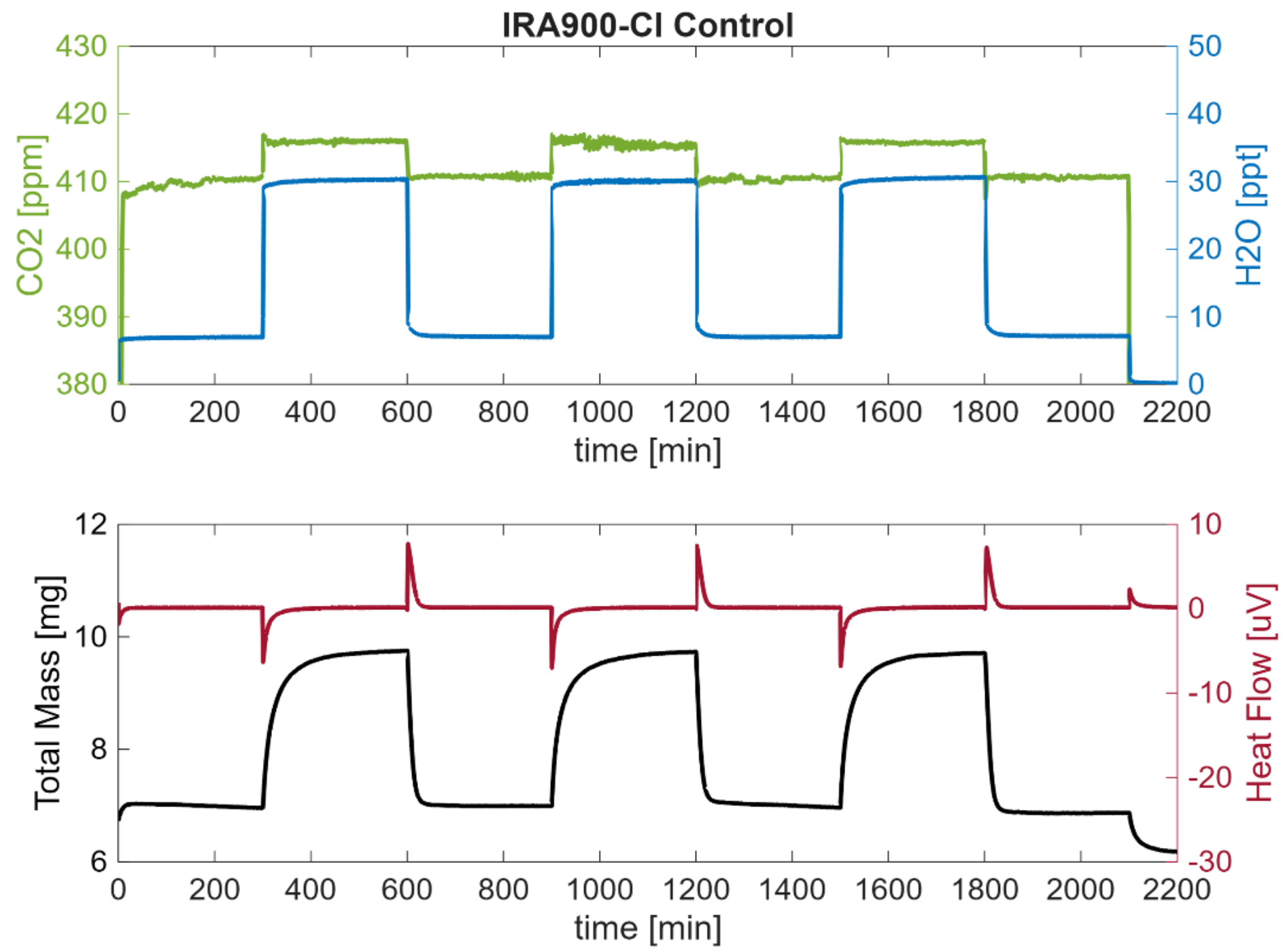

Figure S2. *Control measurements of the MS experiment for IRA900-Cl. The gas analyzer detected* CO_2 *(green) and* H_2O *(blue) concentrations evolved from the outlet of the TGA/DSC instrumentation. No measureable (de)sorption spikes in CO2 upon change in relative humidity were detected, indicating the role of the anion in the MS process.*

Enhancement Factor

The enhancement factor (EF) was calculated based on the bicarbonate vibrational band centered at 1017 cm^{-1} under dry nitrogen (Dry-N_2) conditions. The measured intensity of this peak was 1200 arbitrary units (a.u.) in the presence of the SERS substrate, compared to 200 a.u. in its absence.

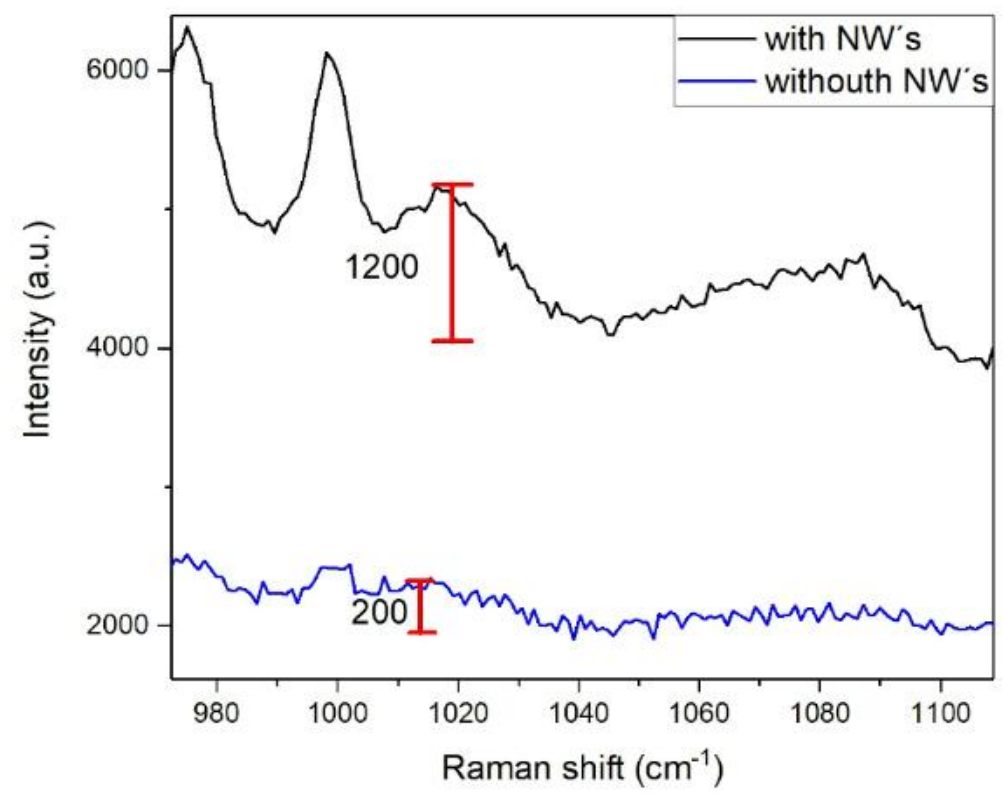

***Figure S3** shows a Raman Intensity measurements of* $IRA900 - HCO_3$ *with Ag-Ni NWs (black line),* $IRA900 - HCO_3$ *without Ag@Ni NWs (blue line).*

To calculate the SERS enhancement factor (EF), equation S1 was used:

$$EF = \frac{I_{SERS} \cdot n_{Raman}}{I_{Raman} \cdot n_{SERS}} \quad \text{(S1)}$$

where I_{SERS} is the Raman intensity with surface enhancement, I_{Raman} is the Raman intensity without surface enhancement, n_{SERS} represents the number of molecules adsorbed on the SERS substrate within the probed area, while n_{Raman} denotes the number of molecules present within the laser's excitation volume during standard Raman scattering measurements.[59-61]

We assume that the laser area is determined by a circle with a radius (r_{laser}) of 21 µm.[49]

$$A_{laser} = \pi r_{laser}^2 = 1.4x10^9 nm^2 \text{ (S2)}$$

The Ni coated Ag nanowire (NW) has a radius ($r_{Ni\ coated\ Ag\ NW}$) of 40 nm and a length ($h_{Ag@Ni\ NW}$) of 4 µm. The cross-sectional area is calculated as follows.

$$A_{NW-cross-section} = 2\pi rh + 2\pi r^2 = 4.0x10^7\ nm^2 \text{ (S3)}$$

The number of Ni coated Ag nanowires ($n_{Ni\ coated\ Ag\ NWs}$) interacting with the laser can be represented as:

$$n_{Ag@Ni\ NWs} = \frac{A_{laser}}{A_{NW\ cross-section}} = \frac{1.4x10^9 nm^2}{4.0x10^7 nm^2} = 34.3 \text{ (S4)}$$

The total surface area of the Ni coated Ag NWs interacting with the laser ($A_{plasmonics}$) can be represented as:

$$A_{plasmonics} = n_{NWs} A_{NW-cross-section} = (4.0x10^7)(34.3) = 1.4x10^9 nm^2 \text{ (S5)}$$

Assuming that the surface area of $IRA900 - HCO_3$ ($A_{IRA900-HCO3}$) is $0.1346\ nm^2$, where the value of r (2.07 Å) was derived from a computational model.[63-64]

$$n_{SERS} = \frac{A_{plasmonics}}{A_{IRA900-HCO3}} = \frac{1.38x10^9 nm^2}{0.1346\ nm^2} = 1.0x10^{10} \text{ (S6)}$$

To calculate the volume of the laser, we assume that the laser has a cylindrical morphology with a height of $h_{laser} = 17.7mm = 1.7x10^7 nm$ [65]

$$V_{laser} = \pi r_{laser}^2 h = \pi(21000nm)^2(1.7x10^7 nm) = 2.4x10^{16} nm^3$$

$$V_{laser} = 2.4x10^{-5} ml \text{ (S7)}$$

We assume that $IRA900 - HCO_3$ has a bicarbonate charge density ($M_{IRA900-HCO3}$) of 0.003 mol/g and a density ($\rho_{IRA900-HCO3}$) of 1.06 g/cm³ to calculate the number of moles ($n_{IRA900-HCO3}$) interacting with the laser

$$n_{IRA900-HCO3} = \frac{V_{laser} \cdot \rho_{IRA900-HCO3}}{M_{IRA900-HCO3}} = \frac{2.4x10^{-5} ml \cdot 1.06\ ^{g}/_{ml}}{0.003\ ^{mol}/_{g}}$$

$$n_{IRA900-HCO3} = 8.5x10^{-3}\ moles \text{ (S8)}$$

n_{Raman} can be expressed as:

$$n_{Raman} = N_A n_{IRA900-HCO3} = 5.1x10^{21} \text{ (S9)}$$

where N_A is Avogadro's number.

Substituting the values obtained in equation (S1), the enhancement factor (EF) can be expressed as:

$$EF = \frac{I_{SERS} \cdot n_{Raman}}{I_{Raman} \cdot n_{SERS}} = 3.0x10^9 \text{ (S10)}$$

The Ni-coated Ag nanowires synthesized in this study exhibited a SERS enhancement factor of approximately $3x10^9$. This EF is indicative of a highly efficient plasmonic substrate, surpassing typical Ag-based nanostructures, which often demonstrate EFs in the range of $10^6 - 10^8$. The high EF can be attributed to the synergistic effect of the Ag core, providing strong localized surface plasmon resonance (LSPR), and the thin Ni coating, which preserves plasmonic activity while offering chemical stability and facilitating potential catalytic interactions. Such an enhancement factor enables the detection of analytes at ultra-low concentrations, demonstrating the potential of these Ni-coated Ag nanowires for applications in trace molecular detection and biosensing. Compared to conventional SERS substrates, this system combines both high sensitivity and structural robustness, highlighting its relevance for next-generation SERS platforms.

Pre-Processing of Raman and SERS Data for Enhanced Quantitative and Qualitative Analysis

To resolve overlapping spectral contributions and improve peak area estimation, a Gaussian deconvolution method was employed. This involved fitting the spectra with a sum of Gaussian functions, constrained by peak positions and widths based on known vibrational modes. Each Raman band was modeled using a Gaussian function, selected based on the instrumental broadening and intrinsic linewidth of the vibrational features. The total Raman spectrum S(υ) was expressed as:

$$S(\upsilon) = \sum_{i=1}^{n} A_i \, f_i(\upsilon; \upsilon_i, \Gamma_i) \qquad \text{(S11)}$$

where A_i is the amplitude (or area) of the peak, υ is the center wavenumber, Γ_i is the full-width at half-maximum (FWHM), and f_i is the functional form representing the peak profile. Initial guesses for peak parameters were provided based on prior spectral knowledge or visual inspection, and constraints were applied to prevent unphysical solutions. Following convergence of the fitting algorithm, the area under each fitted peak A_i, which is proportional to the number of scattering centers contributing to that vibrational mode, was calculated. These peak areas were then used to compute relative intensities by normalizing each area to the total area of all resolved peaks or to a reference peak of known stability, typically as follows :

$$\text{Relative Intensity } \left(I_i^{rel}\right) = \frac{A_i}{\sum_{j=1}^{n} A_j} \qquad \text{(S12)}$$

The deconvolution methodology described above was similarly applied to quantify the intensity of the hydroxyl (–OH) stretching band in the Raman spectra. Following baseline correction and spectral smoothing, the broad –OH band—typically located in the 3100–3600 cm^{-1} region—was fitted using the same nonlinear least-squares approach. A Voigt or Gaussian line shape was selected depending on the spectral broadening and overlap characteristics observed in this region.

O-H stretching deconvolution 0f AC-$KHCO_3$ in hydrated air

The Raman spectra of the AC–$KHCO_3$ sample were analyzed in the O–H stretching region (2800–3800 cm^{-1}) to investigate the evolution of hydroxide anion and hydrogen-bonding environments as a function of time. **Figure S4** displays the deconvolution of spectra collected after (a) 1 h and (b) 2 h of exposure. Prior to peak fitting, the raw spectra were subject to baseline correction to remove fluorescence background, after which a multi-Gaussian fitting procedure was applied. Seven Gaussian components were required to accurately reproduce the broad and asymmetric envelope characteristic of O–H stretching vibrations. Each colored component corresponds to a specific vibrational mode associated with different molecular interactions involving water and hydroxyl species:

- 2879 cm^{-1} (red): Attributed to hydroxyl groups directly hydrogen-bonded to hydroxide ions (OH^-–OH H-bonding).
- 3010 cm^{-1} (green): Assigned to double acceptor–acceptor (DAA) type hydrogen bonding in water.
- 3271 cm^{-1} (purple): Corresponds to double donor–double acceptor (DDAA) water configurations, typical of bulk-like tetrahedral water structures.

- 3305 cm^{-1} (orange): A broad peak representing general hydrogen-bonded species, indicating structural diversity in water networks.
- 3449 cm^{-1} (cyan): Associated with donor–acceptor (DA) water interactions, likely interfacial or disrupted H-bond networks.
- 3576 cm^{-1} (brown): Reflects DDA-type hydrogen bonding involving hydroxyl groups (DDA–OH).
- 3622 cm^{-1} (yellow): Represents non-hydrogen-bonded (free) hydroxide ions (OH^-), often present in more hydrophobic or interfacial regions.

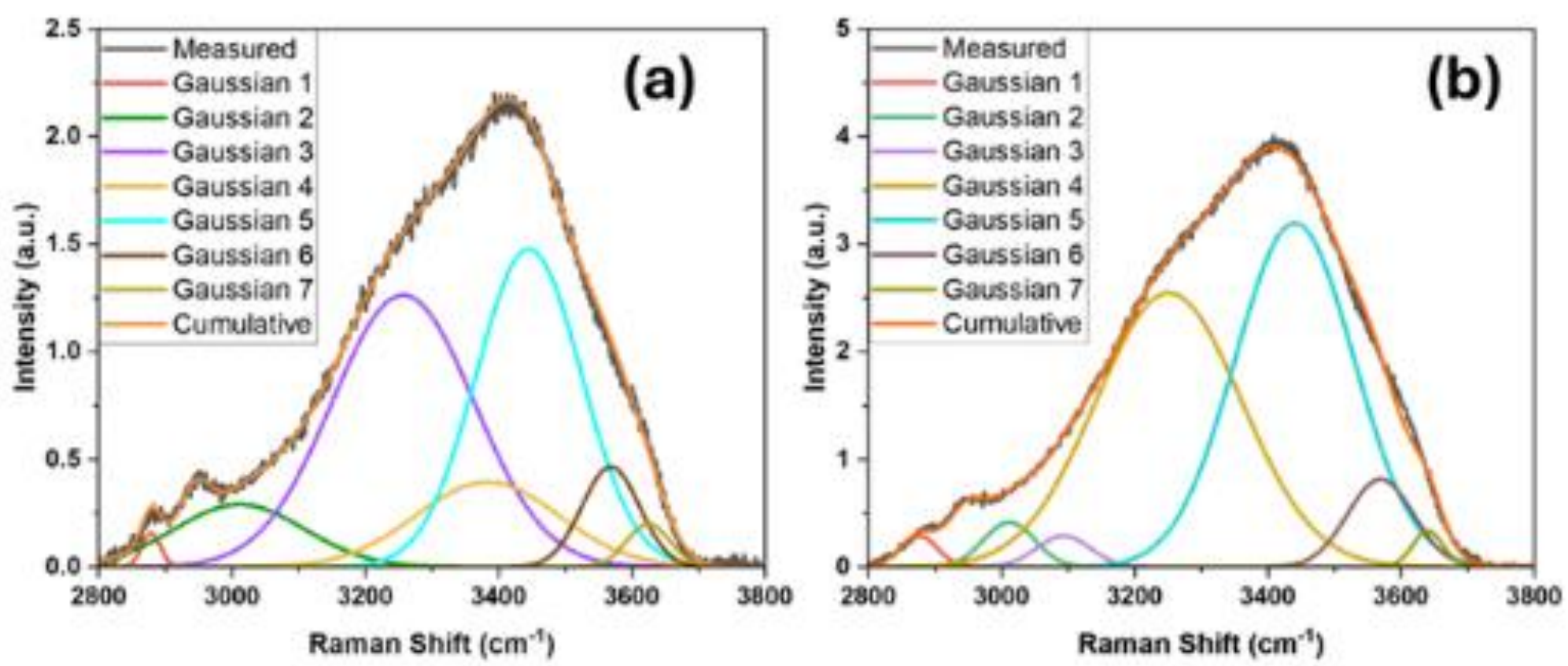

Figure S4. *Deconvolution of the O–H stretching region (2800–3800 cm^{-1}) of Raman spectra for activated carbon–$KHCO_3$. Panel (a) shows the spectrum after 1 h, where seven Gaussian components reveal dominant contributions from strongly H-bonded OH groups near 3200–3400 cm^{-1}. Panel (b) displays the spectrum after 2 h, with increased overall intensity and redistribution toward intermediate OH components at ~3300–3450 cm^{-1}, consistent with enhanced adsorption and reorganization of surface hydroxyl species.*

Table S1. **S**pectral peak positions and their corresponding assignments of the complex hydrogen bonding environments present in the system under study. This spectral decomposition highlights the co-existence of multiple hydrogen-bonded species, crucial for understanding water structure and dynamics in heterogeneous or confined systems.[44]

**Our experimental result peak (cm^{-1})**	**Assignment**
2879	OH H-bonded directly to OH−
3010	DAA water
3271	DDAA water
3305	H bonded, broad peak
3449	DA water
3576	DDA-OH

C-O stretch deconvolution of AC-$KHCO_3$ in dry air

Figure S5 shows the Gaussian deconvolution of a Raman spectral band recorded for the AC-$KHCO_3$ sample under dry-air conditions in the spectral region between 1000 and 1100 cm^{-1}. The measured spectrum (black line) exhibits a single prominent asymmetric peak centered near 1030 cm^{-1}, which has been deconvoluted into three Gaussian components:

- Gaussian 1 (red): A lower-frequency shoulder located around 1019 cm^{-1}, likely associated with bicarbonate-related vibrations or adsorbed species weakly interacting with the carbon matrix.
- Gaussian 2 (blue): The dominant component centered approximately at 1029 cm^{-1}, corresponding to the symmetric stretching mode of bicarbonate anions.
- Gaussian 3 (gold): A smaller high-frequency contribution near 1041 cm^{-1}, potentially arising from structurally distinct or less strongly bound bicarbonate species.

The cumulative fit (purple line) closely matches the experimental data, confirming a good fit of the individual components and indicating that multiple chemically or structurally distinct surface species are present. The spectral profile under dry-air conditions suggests that carbonate and bicarbonate species are retained on the activated carbon surface, possibly through strong chemical interactions or residual moisture effects.

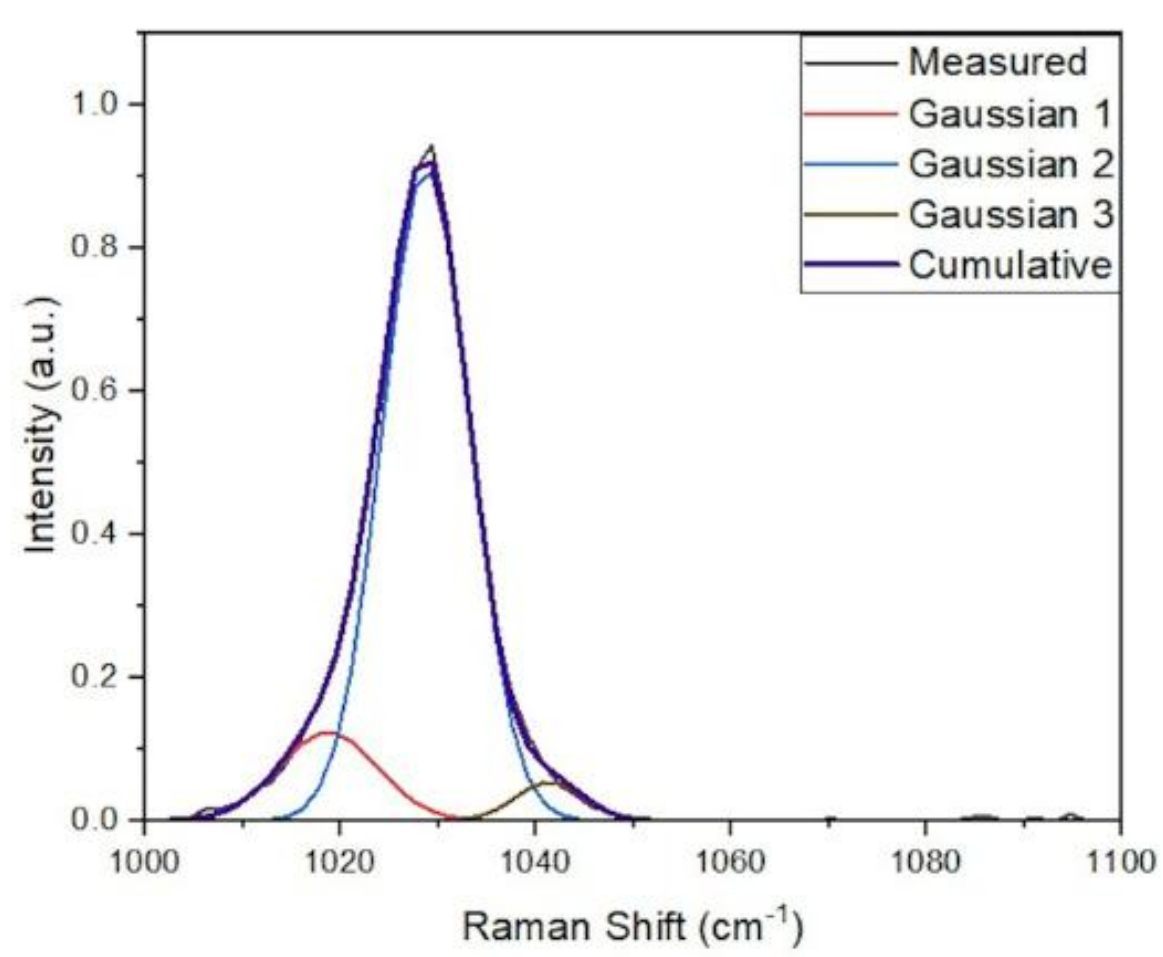

***Figure S5.** shows the Gaussian deconvolution of a Raman spectral band recorded for the AC-$KHCO_3$ sample under dry-air conditions in the spectral region between 1000 and 1100 cm^{-1}. The dominant peak is the bicarbonate anion, the presence of carbonate is not detected.*

Table S2 summarizes the spectral peak positions and their corresponding heights for the AC-$KHCO_3$ sample under dry-air conditions in the spectral region between 1000 and 1100 cm^{-1}.

**Our experimental result peak (cm^{-1})**	**Max height**
1019	0.12537
1029	0.95149
1041	0.06256

O-H stretching deconvolution 0f AC-$KHCO_3$ in humidified N_2

Figure S6. presents the Gaussian deconvolution of the OH stretching region in the Raman spectrum of the AC-$KHCO_3$ sample under humid-N_2 conditions, highlighting the different hydrogen bonding environments of water molecules adsorbed on the surface. The experimental spectrum (black line) spans the range from approximately 2800 to 3800 cm^{-1}, where water-related vibrational modes dominate.

The spectrum has been deconvoluted into six Gaussian components (colored curves), corresponding to distinct OH vibrational environments:

- 2864 cm^{-1} (red): Assigned to OH groups hydrogen-bonded directly to OH^- species, indicating strong interactions with surface hydroxyls.

- 3079cm^{-1} (blue): Corresponds to DAA (Donor-Acceptor-Acceptor) water configurations, indicative of weakly bound water.

- 3165 cm^{-1} (green): Associated with DDAA (Donor-Donor-Acceptor-Acceptor) water, representative of tetrahedrally coordinated hydrogen bonding, resembling bulk-like water clusters.

- 3307 cm^{-1} (purple): A broad band typical of strongly hydrogen-bonded OH groups, often considered a convolution of multiple species and surface environments.

- 3458 cm^{-1} (orange): Attributed to DA (Donor-Acceptor) water species, which interact moderately with the surface or other water molecules.

- 3706 cm^{-1} (cyan): Corresponds to free OH^- stretching vibrations, representing non-hydrogen-bonded OH groups, usually at the surface or interface.

The cumulative fit (brown line) closely overlaps with the measured spectrum, validating the accuracy of the deconvolution. This detailed spectral decomposition reveals a complex

hydrogen bonding network under humid conditions, suggesting the coexistence of both strongly and weakly bound water molecules. The predominance of features around 3160–3300 cm^{-1} highlights the presence of structured water layers or clusters interacting with the functionalized surface of the activated carbon.

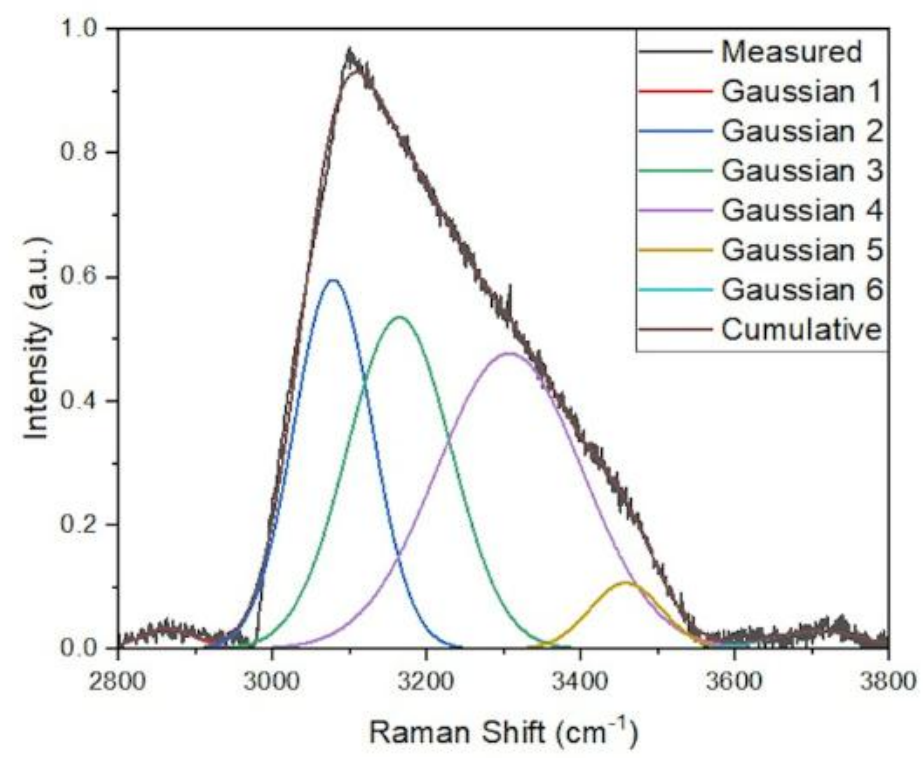

Figure S6. *presents the Gaussian deconvolution of the OH stretching region in the Raman spectrum of the AC-$KHCO_3$ sample under humid-N_2 conditions. Hydrogen bonded hydroxide anions are deconvoluted from the water signal.*

Table S3 summarizes the O-H stretch spectral peak positions and their corresponding assignments, providing insights into the complex hydrogen bonding environments present in the system under study. This spectral decomposition highlights the coexistence of multiple hydrogen-bonded species, crucial for understanding water structure and dynamics in heterogeneous or confined systems.

**Our experimental result peak (cm^{-1})**	**Assignment**
2864	OH H-bonded directly to OH−
3079	DAA water
3165	DDAA water
3307	H bonded, broad peak
3458	DA water
3706	Free OH^{-}

C-O stretching deconvolution 0f AC-$KHCO_3$ in humidified N_2

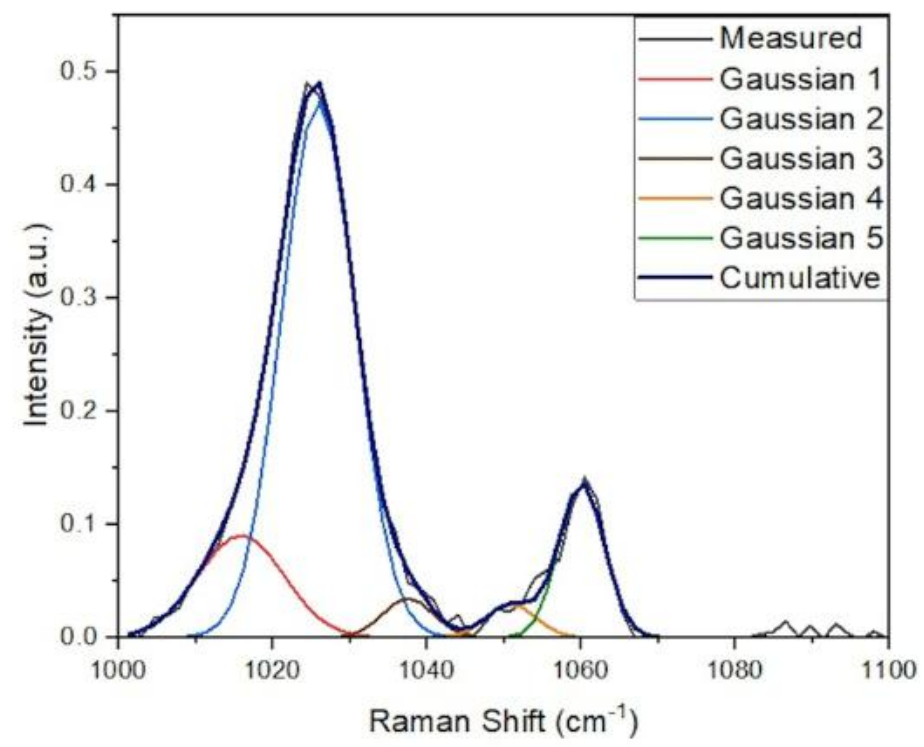

Figure S7. *displays the vibrational fingerprint of the AC-$KHCO_3$ sample in the 1000–1100 cm^{-1} region under dry-N2 conditions.*

Figure S7. displays the vibrational fingerprint of the AC-$KHCO_3$ sample under dry-N2 conditions in the 1000–1100 cm^{-1} region. The black curve represents the measured Raman intensity, which has been deconvoluted into five Gaussian components:[62]

- Gaussian 1 (red) and Gaussian 2 (light blue) contribute primarily to the intense band centered around ~1015–1030 cm^{-1}, likely associated with bicarbonate symmetric stretching (HCO_3^-) vibrations.
- Gaussian 3 (brown) and Gaussian 4 (orange) appear in the 1040–1060 cm^{-1} region, indicating possible overlapping modes or structural perturbations, perhaps due to interactions with the activated carbon matrix.
- Gaussian 5 (green), though minor, contributes around 1055–1060 cm^{-1}, potentially from less intense vibrational features or residual species.

The cumulative fit (dark blue line) closely matches the measured data, validating the multipeak fitting approach.

Table S4 summarizes the spectral peak positions and their corresponding heights for the AC-$KHCO_3$ sample under dry-N_2 conditions in the spectral region between 1000 and 1100 cm^{-1}.

Our experimental result peak (cm^{-1})	Max height
1016	0.09045
1026	0.4797
1038	0.05471
1051	0.04616
1060	0.14732

References

(44) Wakolo, S. W.; Tryk, D. A.; Nishiyama, H.; Miyatake, K.; Iiyama, A.; Inukai, J. Various States of Water Species in an Anion Exchange Membrane Characterized by Raman Spectroscopy under Controlled Temperature and Humidity. *Phys. Chem. Chem. Phys.* **2024**, *26* (3), 1658–1670.

(49) Hernandez Cedillo, A.; Velazquez Salazar, J. J.; Mendez Lozoya, J.; Agyei-Mensah, J. A.; Guirado-López, R. A.; Hernandez Cedillo, C.; Lehr, A.; Yacaman, M. J. *J. Phys. Chem. C* **2025**, *129* (8), 4113–4125.

(59) Xu, Y.; Konrad, M. P.; Trotter, J. L.; McCoy, C. P.; Bell, S. E. J. Rapid One-Pot Preparation of Large Freestanding Nanoparticle–Polymer Films. *Small* **2017**, *13* (2).

(60) Tian, C.; Ding, C.; Liu, S.; Yang, S.; Song, X.; Ding, B.; Li, Z.; Fang, J. Nanoparticle Attachment on Silver Corrugated-Wire Nanoantenna for Large Increases of Surface-Enhanced Raman Scattering. *ACS Nano* **2011**, *5* (12), 9442–9449.

(61) Saminy, P. P.; Sonia, S.; Jayram, N. D.; Shenbagamoorthi, S. Unlocking the Power of Nano Petals: Magnifying Rhodamine 6G Detection Sensitivity in SERS. *Inorg. Chem. Commun.* **2024**, *165*, 112521.

(62) Rudolph, W.; Duguay, M. A.; Ashkin, A. Resonant Optical Second Harmonic Generation in Lithium Iodate. *Opt. Commun.* **1968**, *3*, 29–33.

(63) Hou, C.; Ding, L.; Lai, Z.; Chen, J.; Dong, H.; Lv, H.; Zhang, K.; Wang, T.; Fang, M. Thermodynamic Analysis of Electric Field Enhanced CO_2 Capture by Moisture-Sensitive Quaternary Ammonium-Based Sorbents. *Therm. Sci.* **2024**, *28*, 91–91.

(64) Shi, X.; Xiao, H.; Liao, X.; Armstrong, M.; Chen, X.; Lackner, K. S. Humidity Effect on Ion Behaviors of Moisture-Driven CO_2 Sorbents. *J. Chem. Phys.* **2018**, *149* (16), 164708.

(65) Murphy, D. B.; Davidson, M. W. *Fundamentals of Light Microscopy and Electronic Imaging*, 2nd ed.; Wiley-Blackwell: Hoboken, NJ, 2013.
